# Meta‐analysis on the interrelationship between sarcopenia and mild cognitive impairment, Alzheimer's disease and other forms of dementia

**DOI:** 10.1002/jcsm.13485

**Published:** 2024-05-07

**Authors:** Nadjia Amini, Mounir Ibn Hach, Laurence Lapauw, Jolan Dupont, Laura Vercauteren, Sabine Verschueren, Jos Tournoy, Evelien Gielen

**Affiliations:** ^1^ Gerontology & Geriatrics, Department of Public Health and Primary Care KU Leuven Leuven Belgium; ^2^ Faculty of Medicine KU Leuven Leuven Belgium; ^3^ Department of Geriatric Medicine UZ Leuven Leuven Belgium; ^4^ Research Group for Musculoskeletal Rehabilitation, Department of Rehabilitation Sciences KU Leuven Leuven Belgium

**Keywords:** Alzheimer's disease, cognition, dementia, meta‐analysis, mild cognitive impairment, muscle mass, muscle strength, older adults, physical performance, sarcopenia, systematic review

## Abstract

Sarcopenia has been associated with adverse health outcomes, including cognitive dysfunction. However, its specific interrelationship with neurocognitive disorders such as mild cognitive impairment (MCI), Alzheimer's disease (AD) or other types of dementia has not been thoroughly explored. This meta‐analysis aims to summarize the existing evidence on this interrelationship. This systematic review was pre‐registered on PROSPERO (CRD42022366309) and reported according to the Preferred Reporting Items for Systematic Reviews and Meta‐Analyses 2020 guidelines. Databases, including PubMed, Embase, CINAHL, Scopus, Web of Science, PEDro, SPORTDiscus and the Cochrane Central Register of Controlled Trials, and the data registry ClinicalTrials.gov were searched from inception to 8 June 2023. Observational studies (cross‐sectional and cohort) and interventional studies reporting on the association and prevalence of sarcopenia in MCI, AD or other types of dementia in adults ≥50 years were included. For the meta‐analysis, pooled odds ratios (OR) with 95% confidence intervals (CI) were calculated for the association of sarcopenia with the neurocognitive disorders using random‐effects/fixed‐effects models. Subgroup analyses were performed to identify potential sources of heterogeneity. A total of 77 studies consisting of 92 058 subjects were finally included in the qualitative analysis (71 cross‐sectional, 4 cohort and 2 interventional studies). Studies were heterogeneous, using different diagnostic criteria to define both sarcopenia and cognitive status. The majority of studies (*n* = 38) included Asian community‐dwelling older adults. Most studies investigated the association of sarcopenia with AD (33/77) and MCI (32/77). For studies focusing on other forms of dementia, two studies included Lewy body dementia and one study included Parkinson's dementia, whereas the remaining studies did not specify dementia aetiology (*n* = 21). Three cohort studies explored the association between sarcopenia and incident MCI, whereas only one cohort study explored the association between dementia and incident sarcopenia. Two interventional studies investigated whether an exercise programme could prevent the progression of sarcopenia in older adults with dementia or AD. The information for the meta‐analysis was extracted from 26 studies. Sarcopenia was significantly associated with MCI (pooled OR = 1.58, 95% CI 1.42–1.76) (*n* = 14), AD (pooled OR = 2.97, 95% CI 2.15–4.08) (*n* = 3) and non‐AD dementia (pooled OR = 1.68, 95% CI 1.09–2.58) (*n* = 9). The significance and magnitude of the associations differed in subgroup analyses by study design, population, definition of sarcopenia or used tool to measure cognitive status. This meta‐analysis showed that sarcopenia is significantly associated with MCI, AD and other types of dementia. These findings suggest the importance of early screening and prevention of sarcopenia in older people with cognitive dysfunction, although further longitudinal research is needed to clarify the causal relationship.

## Introduction

Sarcopenia, characterized by the age‐related loss of skeletal muscle mass (SMM) and function, is a prevalent condition among older adults.^1^ A recent meta‐analysis has found that the prevalence of sarcopenia ranges between 10% and 27% in individuals over the age of 60 years. This heterogeneous prevalence rate can be attributed to the use of diverse diagnostic criteria, variability in measurement techniques and cut‐off values, as well as different study populations.^2^ Sarcopenia is a condition with a multifactorial aetiology, including chronic inflammation, hormone depletion, malnutrition and physical inactivity, and is linked with adverse health outcomes such as falls and fractures, disability and mortality.^3^, ^4^

Another key feature of the ageing process is cognitive dysfunction. For instance, mild cognitive impairment (MCI) is defined as the intermediate stage between normal age‐related cognitive decline and dementia and affects up to 19% of adults over the age of 65 years.^5^ Individuals with MCI have cognitive dysfunctions that go beyond what is expected during the normal ageing process, yet the level of dysfunction is not severe enough to hinder the activities of daily living (ADLs).^6^ It is stated that within 4–6 years, more than 50% of older adults with MCI progress to dementia.^7^ Moreover, Alzheimer's disease (AD) is 10 times more likely to occur in those with MCI. The most common type of dementia is AD, followed by vascular dementia, dementia with Lewy bodies, frontotemporal dementia and Parkinson's dementia.^6^ Dementia is reported to affect 7.1–16.3% of adults over the age of 65 years and has a substantial financial impact on health care expenditures, in addition to a high personal and social burden. Thus, early detection and management of MCI are crucial in order to decrease the progression of the disease and delay the onset of dementia.^8^

Emerging evidence suggests a potential relationship between sarcopenia and cognitive dysfunction, such as MCI, AD and other forms of dementia.^9^, ^10^, ^11^, ^12^ Several mechanisms have been hypothesized to explain the association between sarcopenia and cognitive disorders. Possible explanations involve shared pathways of inflammation, oxidative stress, insulin resistance and hormonal changes, as well as shared common risk factors such as physical inactivity and malnutrition.^13^

Sarcopenia and its relationship with cognitive impairment have been investigated and quantified in several systematic reviews and meta‐analyses.^9^, ^10^, ^13^ However, in recent years, there has been a substantial growth of studies published on this topic, which warrants an up‐to‐date systematic review and meta‐analysis. Clarifying the link between sarcopenia and cognitive decline could lead to more effective planning for dementia prevention and treatment.

In addition, most previous systematic reviews exploring this relationship have focused on the association between sarcopenia and cognitive impairment without further specification, so this may include individuals with mild, moderate and severe dementia. As a result, the relationship between sarcopenia and MCI, AD and other forms of dementia as separate conditions remains unclear.^9^, ^10^, ^13^

Therefore, this meta‐analysis aims to summarize and quantify the existing evidence on the interrelationship between sarcopenia and cognitive dysfunction, with a focus on MCI, AD and other forms of dementia. In addition, the second aim is to examine whether study characteristics or participant characteristics modify the relationship between both conditions in subgroup analysis.

## Methodology

### Search strategy and study selection

This systematic review is reported according to the Preferred Reporting Items for Systematic Reviews and Meta‐Analyses 2020 (PRISMA 2020) guidelines and pre‐registered on PROSPERO (CRD42022366309). A comprehensive search was performed in eight databases, including MEDLINE via PubMed, Embase, Cochrane Library, CINAHL, PEDro, Scopus, SPORTDiscus and Web of Science, and the data registry ClinicalTrials.gov. The search included a combination of Medical Subject Headings (MeSH) terms with related free text terms/keywords (‘sarcopenia’, ‘mild cognitive impairment’, ‘Alzheimer's disease’ and ‘dementia’) from inception to 8 June 2023. The detailed search strategies for all included databases can be found in *Table* *S1*. Additional articles were identified by citation searching in reference lists of relevant original studies or reviews.

Inclusion criteria for eligible articles were as follows: (1) human observational studies (cohort, case–control and cross‐sectional) and randomized clinical trials; (2) adults aged 50 years and older (due to the atrophy of the muscle and steadily decline in cognitive performance from this age on)^14^, ^15^, ^16^, ^17^; (3) living in the community, residential‐living or hospitalized; and (4) study population involving persons with sarcopenia and persons with MCI, AD or other forms of dementia. Exclusion criteria were as follows: (1) persons suffering from an underlying neurological or neurodegenerative condition primarily affecting the muscle (e.g., polyneuropathy, amyotrophic lateral sclerosis and multiple sclerosis); (2) animal studies, case series or reports, literature reviews, conference abstracts, studies of in vitro models, protocols without results, letters to editors and studies not reporting in English, Dutch, French or German; and (3) number of persons diagnosed with sarcopenia or cognitive dysfunction not reported.

After deduplication, titles and abstracts of all databases were screened independently by two reviewers (NA and MIH), followed by a full‐text review for eligibility. When relevance could not be determined from the abstract, the full‐text paper was retrieved and reviewed. Any disagreements were resolved by a third reviewer (EG).

Data were extracted using a standardized data abstraction form, including the first author's last name, publication year, country, study design, study population, sample size, average age or age category, gender distribution, methods of evaluating sarcopenia and the cognitive outcomes and the main findings related to the prevalence and the association of sarcopenia and cognitive decline. The cognitive outcomes were classified into MCI, AD and non‐AD dementia, depending on how the authors reported these cognitive outcomes in the included studies.

### Quality assessment

The quality of each study was assessed and scored by two independent reviewers (NA and MIH) using the Newcastle–Ottawa Scale (NOS; eight‐item, maximum score: 9) for cohort studies, the adapted NOS for cross‐sectional studies (NOS; six‐item, maximum score: 7) and the revised Cochrane risk‐of‐bias tool for randomized trials (RoB2) and the Risk Of Bias In Non‐Randomized Studies of Interventions (ROBINS‐I) tool for non‐randomized trials. When agreement was not met, a third reviewer (EG) was consulted for a final solution.

### Statistical analyses

The main outcome of interest was the (adjusted) association between sarcopenia and MCI, AD and other forms of dementia, expressed in odds ratios (ORs) and 95% confidence intervals (CIs). Summary estimates and corresponding 95% CIs for the outcome were pooled. Results were analysed separately when a study included multiple sarcopenia assessment methods or reported results of different stages in cognitive dysfunction or sarcopenia severity. Random‐effects models were used in the meta‐analysis if the *I* ^2^ test detected significant heterogeneity (*χ* ^2^‐based Cochran's *Q* statistic test and *I* ^2^ statistic: *I* ^2^ > 50, *P* < 0.05). Otherwise, fixed‐effects models were applied. Subgroup analyses by study design, study population, sarcopenia assessment, cognitive status assessment, gender, study region and study quality were performed to identify potential sources of heterogeneity. Sensitivity analyses were also performed to determine the quality and robustness of the results by deleting one study at a time (*Table* *S4*). Lastly, Egger's test (significance level *P* < 0.10) and funnel plots were applied to determine potential publication bias (*Figure* *S1* and *Table* *S5*). All statistical analyses were performed using the meta package in Stata, Version 17.0 (StataCorp LLC, TX, USA). All results were considered significant if *P* < 0.05.

## Results

### Study selection and characteristics of included population

**Figure 1:**
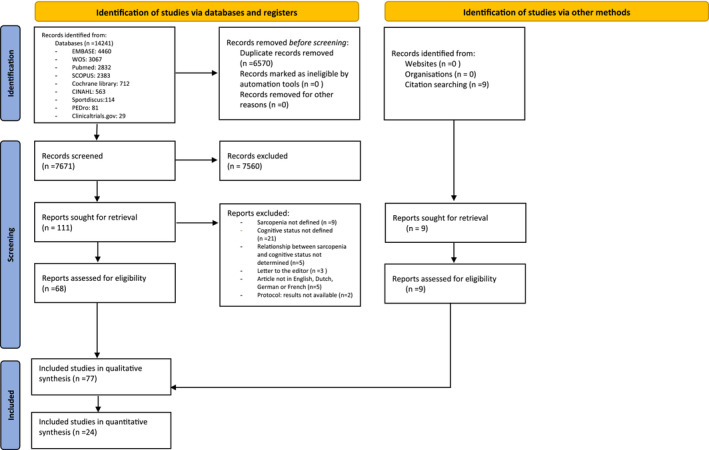
Flowchart of the study search. WOS, Web of Science.

The flow diagram of the literature retrieval process is shown in *Figure* *1*. Seventy‐seven studies with a sample size ranging from 26 to 12 912 participants and a total of 92 058 participants were included in the qualitative analysis.^11^, ^12^, ^18^, ^19^, ^20^, ^21^, ^22^, ^23^, ^24^, ^25^, ^26^, ^27^, ^28^, ^29^, ^30^, ^31^, ^32^, ^33^, ^34^, ^35^, ^36^, ^37^, ^38^, ^39^, ^40^, ^41^, ^42^, ^43^, ^44^, ^45^, ^46^, ^47^, ^48^, ^49^, ^50^, ^51^, ^52^, ^53^, ^54^, ^55^, ^56^, ^57^, ^58^, ^59^, ^60^, ^61^, ^62^, ^63^, ^64^, ^65^, ^66^, ^67^, ^68^, ^69^, ^70^, ^71^, ^72^, ^73^, ^74^, ^75^, ^76^, ^77^, ^78^, ^79^, ^80^, ^81^, ^82^, ^83^, ^84^, ^85^, ^86^, ^87^, ^88^, ^89^, ^90^, ^91^, ^92^ Of these 77 studies, 71 were cross‐sectional, 4 were cohort and 2 were interventional studies.

Most of the studies focused on sarcopenia and AD (33 studies), whereas 32 studies reported on MCI and 25 studies reported on other forms of dementia. Of these 25 studies, only 3 studies specified the dementia aetiology (2 studies on Lewy body dementia and 1 study on Parkinson's dementia).

Moreover, two studies reported on both MCI and dementia, eight studies reported on both MCI and AD, and two studies reported on both AD and other types of dementia. Eight studies focused solely on the association between sarcopenia and neurocognitive disorders, whereas 44 studies reported on the prevalence of sarcopenia in cognitive dysfunction or vice versa. Twenty‐five studies analysed both the association and prevalence of both conditions.

The majority of the studies were conducted in Asia (45 studies) and Europe (25 studies), whereas the other studies were performed in Australia, Africa and America. Among the included studies, 60 studies were from the community‐dwelling/outpatient clinical setting, 12 studies were conducted with hospitalized patients and 5 studies were conducted in nursing homes or long‐term care institutions. The majority of the studies used the Asian Working Group for Sarcopenia (AWGS1 or AWGS2) criteria (31 studies) and the (revised) European Working Group on Sarcopenia in Older People (EWGSOP1 or EWGSOP2) criteria (31 studies) to diagnose sarcopenia. Six studies used the SARC‐F tool, seven studies used the method of low SMM (self‐defined criteria) or low SMM index (SMI) (Baumgartner definition), and one study used the Foundation for the National Institutes of Health definition (FNIH) to define sarcopenia. Four studies used various other methods to diagnose sarcopenia, such as low muscle thickness (via ultrasound) combined with low grip strength, low calf circumference, the Short Portable Sarcopenia Measure and medical record.

Numerous tools were used to define MCI, including the Mini Mental State Examination (MMSE) (eight studies), the (modified) Petersen criteria (seven studies), the Montreal Cognitive Assessment (MoCA) (five studies), the National Institute on Aging—Alzheimer's Association (NIA‐AA) (five studies) and the Short Portable Mental Status Questionnaire (SPMSQ) (two studies). Seven studies used other tools to diagnose MCI.

To diagnose AD, 11 studies used the criteria of the National Institute of Neurological and Communicative Disorders and Stroke and the Alzheimer's Disease and Related Disorders Association (NINCDS‐ADRDA), 10 studies applied the NIA‐AA criteria and 8 studies used the Diagnostic and Statistical Manual of Mental Disorders (DSM) method. Four studies used other tools to diagnose AD. The majority of the studies used the medical record (five studies), the NIA‐AA criteria (three studies) and the MMSE test (two studies) to diagnose other forms of dementia.

Most of the studies included both genders (70 studies), whereas three studies included only men and four studies included only women. Of the studies providing specific information on age (75 studies) and gender (74 studies), the mean age was 76.3 years and 41.8% of the subjects were men. The adjusted confounders varied between studies, but the majority of the studies (34 studies) adjusted for at least one of the following confounders: age, gender, education, physical activity and number of comorbidities.

### Prevalence of sarcopenia in populations with neurocognitive dysfunction and vice versa

Of the studies reporting on the prevalence of neurocognitive dysfunction in sarcopenia or vice versa, a heterogeneous range of prevalence rates was found. For instance, the prevalence of sarcopenia ranged from 2.8% to 73.9% in older adults with MCI (12 studies), from 4.2% to 86.6% in older adults with AD (30 studies) and from 15.4% to 61.3% in older adults with other types of dementia (10 studies). Vice versa, in older adults with sarcopenia, the prevalence of MCI ranged from 10.5% to 71.0% (12 studies), the prevalence of AD ranged from 23.3% to 62.2% (2 studies) and the prevalence of other forms of dementia ranged from 6.5% to 84.1% (9 studies).

### Results from longitudinal studies

Among the four cohort studies (follow‐up range between 3 and 8 years), three studies reported on the association between sarcopenia and incident MCI or AD. For instance, both Salinas‐Rodríguez et al. and Hu et al. found that having sarcopenia was significantly associated with incident MCI (Salinas‐Rodríguez et al.: OR = 1.74, 95% CI 1.02–2.96 and Hu et al.: OR = 1.72, 95% CI 1.04–2.85), whereas Beeri et al. found that having severe sarcopenia was associated with both higher risk of incident MCI (hazard ratio [HR] = 1.21, 95% CI 1.01–1.45) and AD (HR = 1.57, 95% CI 1.20–1.86).^11^, ^36^, ^61^ On the other hand, only one study explored the relationship from the opposite direction: from cognitive dysfunction to subsequent incident sarcopenia. This study reported that having probable dementia was not significantly associated with incident sarcopenia after 4 years of follow‐up (OR = 0.98, 95% CI 0.65–1.49).

Both interventional studies included in this systematic review investigated whether an exercise programme could prevent progression of loss of muscle mass and strength (i.e., sarcopenia) in older adults with dementia or AD.^38^, ^91^ Of these two interventional studies (intervention period: 12 weeks), one study used a non‐randomized study design and one study used a randomized design.^38^, ^91^ Two different exercise programmes were used in these studies. The exercise programme of the study by Henwood et al. consisted of group swim exercises,^38^ whereas the participants in the study by Yun et al. performed solo balloon exercises in supine position.^91^ Results from these interventional studies showed that there was no significant difference in the prevalence of sarcopenia between exercise group and control group after the 12‐week intervention period in older adults with cognitive dysfunction.

Detailed information extracted from the included studies is summarized in *Table* *S2*.

### Quality assessment

Among the observational studies, 3 studies were of high quality (NOS: ≥7 points), 26 studies were of moderate quality (NOS: 4–6 points) and 46 studies were of low quality (NOS: 0–3 points). The majority of studies were of low quality due to uncertain representativeness of the sample, unsatisfactory or unclear response rates, and the use of non‐validated methods to measure sarcopenia or neurocognitive disorders. The interventional study by Henwood et al. had a serious overall risk of bias, and the other interventional study by Yun et al. had a low overall risk of bias. More detailed information on the quality assessment of the included studies can be found in *Table* *S3*.

### Results of meta‐analyses

#### Association between sarcopenia and mild cognitive impairment

**Figure 2:**
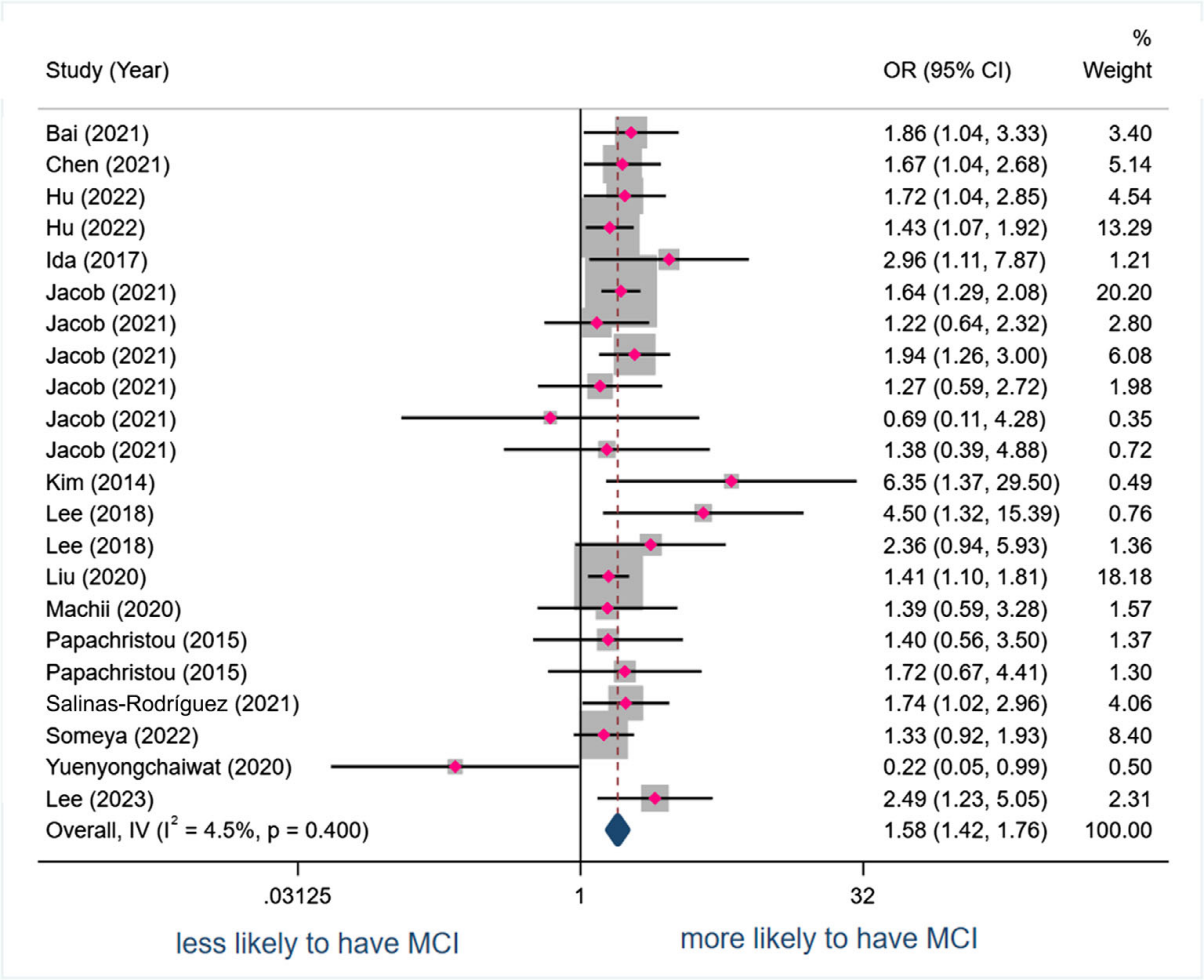
Forest plot of the adjusted odds ratio (OR) between sarcopenia and mild cognitive impairment (MCI). Results from the fixed‐effects model. Hu et al. gave results on two levels of sarcopenia severity with MCI, so this study was classified into two different analyses. Papachristou et al. used two different definitions for sarcopenia, so this study was classified into two different analyses. Jacob et al. assessed the association between sarcopenia and MCI in six different countries, so this study was classified into six different analyses. CI, confidence interval.

A total of 14 studies with 30 241 subjects were included for the calculation of the pooled OR. The pooled adjusted OR between sarcopenia and MCI was 1.58 (95% CI 1.42–1.76) with a low level of heterogeneity (*I* ^2^ = 4.5%; *P* = 0.400) (*Figure* *2*), indicating that a person with sarcopenia has 1.58 more odds of having MCI compared to a person without sarcopenia. The study by Yuenyongchaiwat and Boonsinsukh is the only study that found a negative association between sarcopenia and MCI (OR = 0.22, 95% CI 0.05–0.99).^90^

Results from the subgroup analyses demonstrated that sarcopenia was associated with MCI in studies using the AWGS1 and AWGS2, EWGSOP1 and EWGSOP2, and SARC‐F definitions, but not when the FNIH criteria were used to define sarcopenia. Sarcopenia was associated with MCI in both cross‐sectional and cohort studies. Sarcopenia was not associated with MCI when the MoCA test was used (OR = 1.23, 95% CI 0.88–1.71).

*Table* *1* shows the results of the subgroup analyses of the association between MCI and sarcopenia.

**Table 1:** Subgroup analysis of the association between sarcopenia and MCI

Study characteristic	Number of studies	Results from meta‐analysis	Heterogeneity
Study population			
Community‐dwelling/outpatients	14	1.58 (1.42–1.76)	*I* ^2^ = 4.5%; *P* = 0.400
Study design			
Cross‐sectional	12	1.59 (1.41–1.79)	*I* ^2^ = 15.5%; *P* = 0.264
Cohort	2	1.54 (1.22–1.94)	*I* ^2^ = 0.0%; *P* = 0.730
Sarcopenia assessment			
AWGS1	4	1.50 (1.21–1.85)	*I* ^2^ = 62.5%; *P* = 0.030
AWGS2	5	1.53 (1.27–1.85)	*I* ^2^ = 0.0%; *P* = 0.659
EWGSOP1	3	1.84 (1.18–2.86)	*I* ^2^ = 30.6%; *P* = 0.237
EWGSOP2	1	1.60 (1.33–1.94)	*I* ^2^ = 0.0%; *P* = 0.746
FNIH	1	1.72 (0.67–4.41)	—
SARC‐F	1	2.96 (1.11–7.87)	—
Cognitive status assessment			
MMSE	2	3.43 (1.76–6.67)	*I* ^2^ = 0.0%; *P* = 0.487
MoCA	3	1.23 (0.88–1.71)	*I* ^2^ = 62.2%; *P* = 0.071
Modified Petersen criteria	2	1.74 (1.21–2.52)	*I* ^2^ = 0.0%; *P* = 0.778
TYM	2	1.89 (1.10–3.27)	*I* ^2^ = 0.0%; *P* = 0.7533
SPMSQ	1	1.41 (1.10–1.81)	—
NIA‐AA	2	1.62 (1.35–1.93)	*I* ^2^ = 0.0%; *P* = 0.836
Other	2	1.59 (1.25–2.02)	*I* ^2^ = 6.6%; *P* = 0.343
Gender			
Both genders	12	1.56 (1.39–1.74)	*I* ^2^ = 7.1%; *P* = 0.370
All female	1	2.98 (1.42–6.22)	*I* ^2^ = 0.0%; *P* = 0.410
All male	1	1.55 (0.80–2.99)	*I* ^2^ = 0.0%; *P* = 0.759
Study region			
Asia	12	1.59 (1.42–1.77)	*I* ^2^ = 27.2%; *P* = 0.150
Europe	2	1.51 (0.84–2.71)	*I* ^2^ = 0.0%; *P* = 0.942
Central/South America	2	1.57 (1.01–2.43)	*I* ^2^ = 0.0%; *P* = 0.507
Africa	1	0.69 (0.11–4.28)	—
Study quality			
Low	2	1.64 (1.36–1.97)	*I* ^2^ = 0.0%; *P* = 0.655
Moderate	11	1.57 (1.34–1.83)	*I* ^2^ = 29.9%; *P* = 0.145
High	1	1.50 (1.16–1.93)	*I* ^2^ = 0.0%; *P* = 0.535

Abbreviations: AWGS, Asian Working Group for Sarcopenia; EWGSOP, European Working Group on Sarcopenia in Older People; FNIH, Foundation for the National Institutes of Health definition; MCI, mild cognitive impairment; MMSE, Mini Mental State Examination; MoCA, Montreal Cognitive Assessment; NIA‐AA, National Institute on Aging—Alzheimer's Association; SPMSQ, Short Portable Mental Status Questionnaire; TYM, Test Your Memory.

#### Association between sarcopenia and Alzheimer's disease

**Figure 3:**
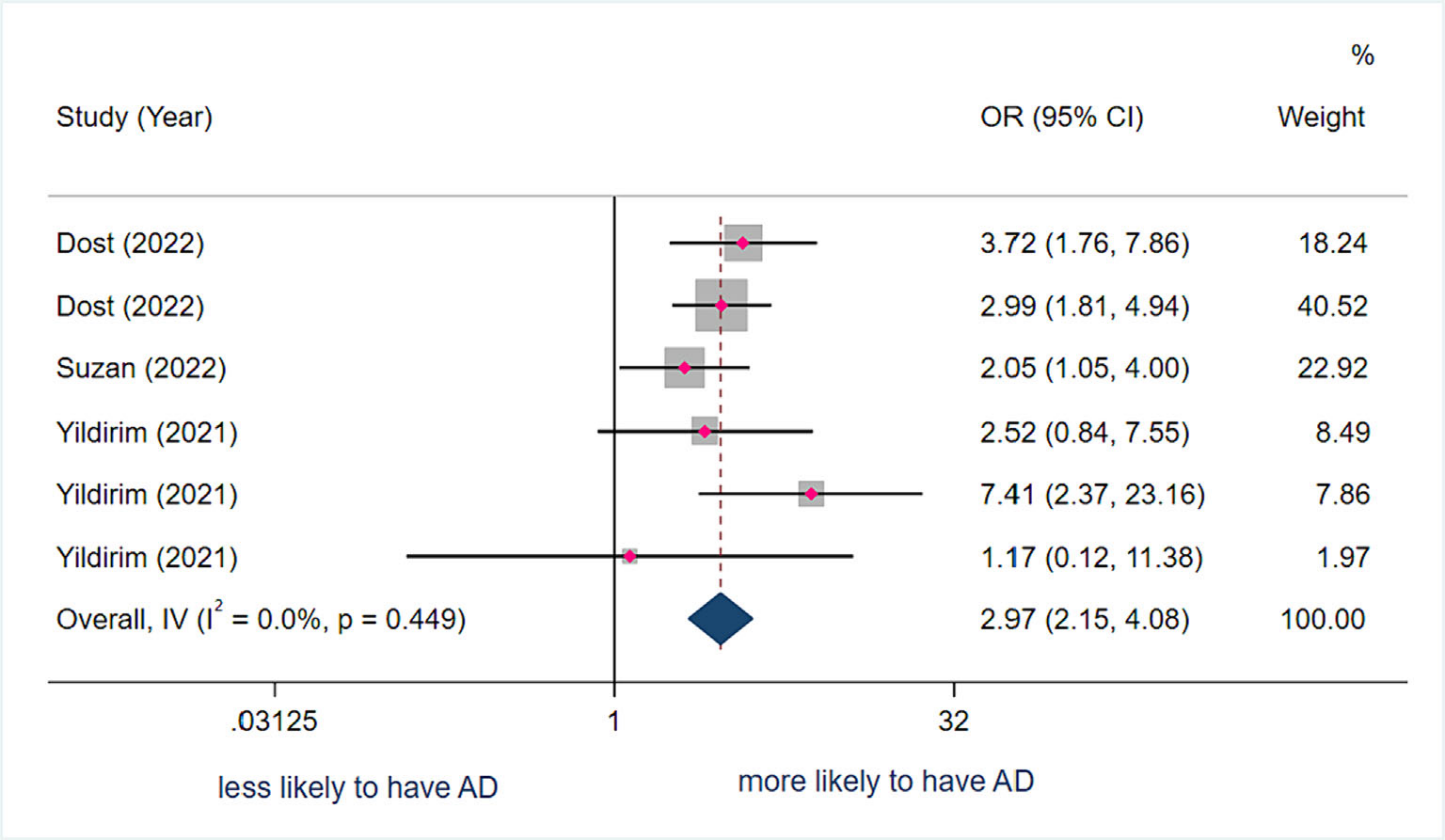
Forest plot of the adjusted odds ratio (OR) between sarcopenia and Alzheimer's disease (AD) dementia. Results from the fixed‐effects model. Dost et al. reported results on two levels of sarcopenia severity with AD, so this study was classified into two different analyses. Yildirim et al. reported results on the association of sarcopenia with three levels of AD severity, so this study was classified into three different analyses. CI, confidence interval.

Three studies with 1631 subjects were included for the calculation of the pooled OR. The pooled adjusted OR between sarcopenia and AD was 2.97 (95% CI 2.15–4.08) (*I* ^2^ = 0.0%; *P* = 0.449), indicating that a person with sarcopenia has 2.97 more odds of having AD compared to a person without sarcopenia (*Figure* *3*).

Results from the subgroup analyses demonstrated that sarcopenia was associated with AD regardless of the study population, study design, or used tool to define sarcopenia and AD.

*Table* *2* shows the results of the subgroup analysis of the association between AD dementia and sarcopenia.

**Table 2:** Subgroup analysis of the association between sarcopenia and Alzheimer's disease

Study characteristic	Number of studies	Results from meta‐analysis	Heterogeneity
Study population			
Community‐dwelling	2	2.66 (1.62–4.38)	*I* ^2^ = 28.5%; *P* = 0.241
Hospitalized persons	1	3.20 (2.11–4.85)	*I* ^2^ = 0.0%; *P* = 0.635
Study design			
Cross‐sectional	3	2.97 (2.15–4.08)	*I* ^2^ = 0.0%; *P* = 0.449
Sarcopenia assessment			
EWGSOP2	2	2.82 (1.98–4.02)	*I* ^2^ = 0.0%; *P* = 0.483
Medical record	1	3.69 (1.75–7.77)	*I* ^2^ = 30.6%; *P* = 0.237
Cognitive status assessment			
NIA‐AA	2	3.731 (2.30–4.76)	*I* ^2^ = 0.0%; *P* = 0.523
DSM‐5	1	2.05 (1.05–4.00)	—
Gender			
Both genders	3	2.97 (2.15–4.08)	*I* ^2^ = 0.0%; *P* = 0.449
Study region			
Europe	3	2.97 (2.15–4.08)	*I* ^2^ = 0.0%; *P* = 0.449
Study quality			
Low	2	2.66 (1.62–4.38)	*I* ^2^ = 28.5%; *P* = 0.241
Moderate	1	3.20 (2.11–4.85)	*I* ^2^ = 0.0%; *P* = 0.635

Abbreviations: DSM‐5, Diagnostic and Statistical Manual of Mental Disorders, Fifth Edition; EWGSOP, European Working Group on Sarcopenia in Older People; NIA‐AA, National Institute on Aging—Alzheimer's Association.

#### Association between sarcopenia and other types of dementia (non‐Alzheimer's disease)

**Figure 4:**
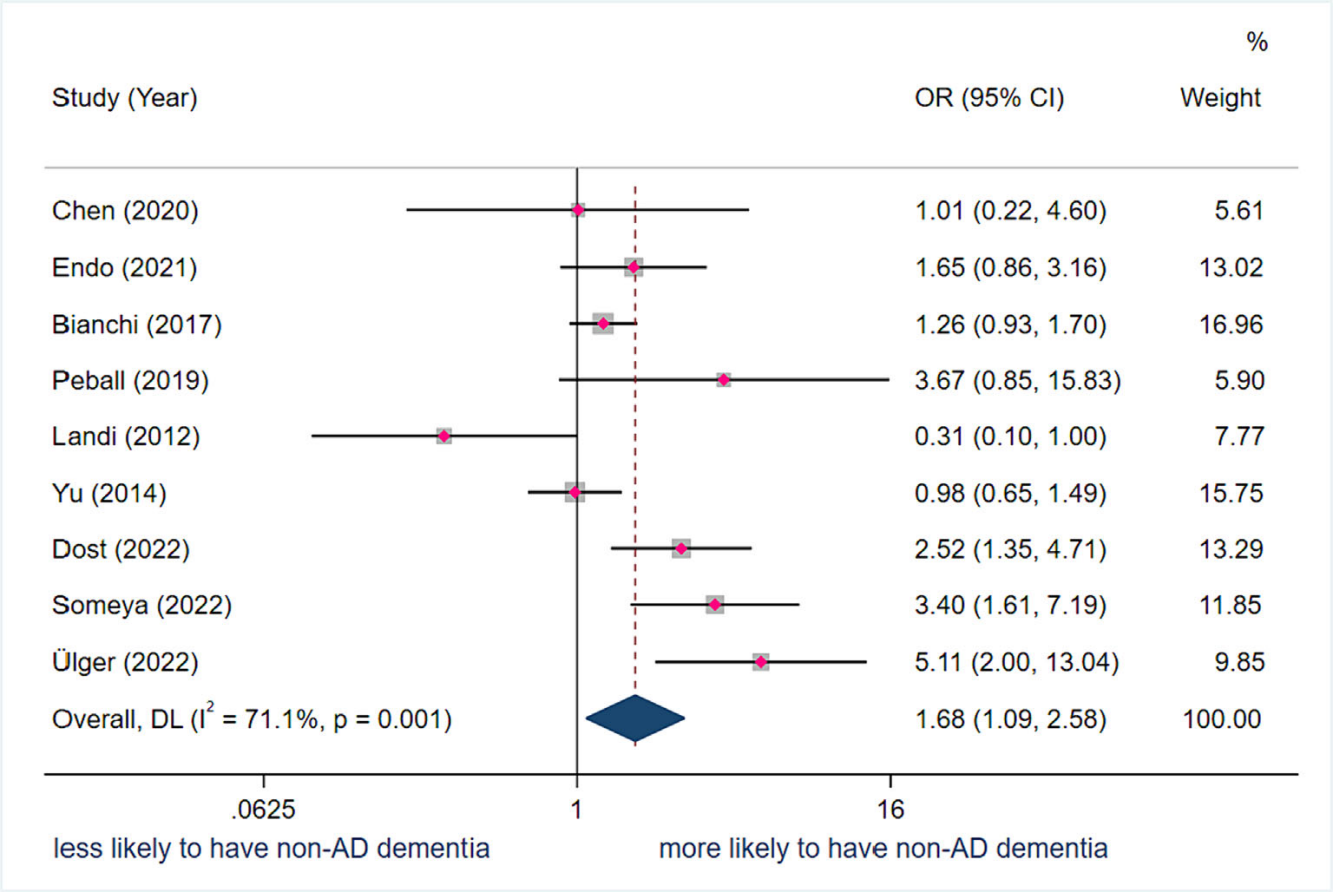
Forest plot of the adjusted odds ratio (OR) between sarcopenia and non‐Alzheimer's disease (AD) dementia. Results from the random‐effects model. CI, confidence interval.

Nine studies with 8601 participants were included for the calculation of the pooled OR. The pooled adjusted OR between sarcopenia and non‐AD dementia was 1.68 (95% CI 1.09–2.58) with a substantial level of heterogeneity (*I* ^2^ = 71.1%; *P* = 0.001), indicating that a person with sarcopenia has 1.68 more odds of having non‐AD dementia compared to a person without sarcopenia (*Figure* *4*). The study by Landi et al. is the only study that found a negative association between sarcopenia and non‐AD dementia (OR = 0.31, 95% CI 0.10–1.00).^47^

Results from the subgroup analyses demonstrated that the association between sarcopenia and other forms of dementia was only significant in EWGSOP2‐diagnosed sarcopenia (OR = 2.52, 95% CI 1.35–4.71), in AWGS2‐diagnosed sarcopenia (OR = 3.40, 95% CI 1.61–7.19) and in the low gastrocnemius muscle thickness‐diagnosed sarcopenia definition (OR = 5.11, 95% CI 2.00–13.04). Moreover, sarcopenia was only associated with other forms of dementia in hospitalized persons (OR = 1.90, 95% CI 1.08–3.34). All cross‐sectional studies found a significant association between sarcopenia and dementia (OR = 1.85, 95% CI 1.13–3.04). Only one cohort study explored the association between dementia and sarcopenia and found that having probable dementia was not significantly associated with incident sarcopenia after 4 years of follow‐up (OR = 0.98, 95% CI 0.65–1.49). Nine different tools were used to define dementia. Only two studies specified the dementia subtype, one study explored Parkinson's dementia and one study focused on Lewy body dementia.

*Table* *3* shows the results of the subgroup analyses of the association between AD and sarcopenia.

**Table 3:** Subgroup analysis of the association between sarcopenia and non‐AD dementia

Study characteristic	Number of studies	Results from meta‐analysis	Heterogeneity
Study population			
Community‐dwelling	3	1.85 (0.79–4.31)	*I* ^2^ = 80.7%; *P* = 0.006
Hospitalized persons	4	1.90 (1.08–3.34)	*I* ^2^ = 65.3%; *P* = 0.034
Other	2	1.03 (0.09–11.54)	*I* ^2^ = 85.0%; *P* = 0.010
Study design			
Cross‐sectional	8	1.85 (1.13–3.04)	*I* ^2^ = 70.0%; *P* = 0.002
Cohort	1	0.98 (0.65–1.49)	—
Sarcopenia assessment			
AWGS1	2	1.53 (0.84–2.78)	*I* ^2^ = 0.0; *P* = 0.560
AWGS2	1	3.40 (1.61–7.19)	—
EWGSOP1	3	0.95 (0.58–1.54)	*I* ^2^ = 63.8%; *P* = 0.063
EWGSOP2	1	2.52 (1.35–4.71)	—
SARC‐F	1	3.67 (0.85–15.83)	—
Low sonographic muscle thickness + low HGS	1	5.11 (2.00–13.04)	—
Cognitive status assessment			
SPMSQ	1	1.01 (0.22–4.60)	—
Medical record	2	0.70 (0.18–2.73)	*I* ^2^ = 74.2%; *P* = 0.021
Fourth consensus report of the Lewy body dementia	1	2.52 (1.35–4.71)	—
MMSE	1	3.40 (1.61–7.19)	—
NIA‐AA and DSM‐5	1	5.11 (2.00–13.04)	—
Other	3	1.41 (0.78–2.53)	*I* ^2^ = 50.8%; *P* = 0.131
Gender			
Both genders	9	1.68 (1.09–2.58)	*I* ^2^ = 71.1%; *P* = 0.001
Study region			
Asia	4	1.56 (0.85–2.87)	*I* ^2^ = 65.1%; *P* = 0.035
Europe	5	1.78 (0.85–3.73)	*I* ^2^ = 78.8%; *P* = 0.001
Study quality			
Low	1	1.01 (0.22–4.60)	—
Moderate	7	1.94 (1.14–3.29)	*I* ^2^ = 73.8%; *P* = 0.001
High	1	0.98 (0.65–1.49)	—

Abbreviations: AD, Alzheimer's disease; AWGS, Asian Working Group for Sarcopenia; DSM‐5, Diagnostic and Statistical Manual of Mental Disorders, Fifth Edition; EWGSOP, European Working Group on Sarcopenia in Older People; HGS, handgrip strength; MMSE, Mini Mental State Examination; NIA‐AA, National Institute on Aging—Alzheimer's Association; SPMSQ, Short Portable Mental Status Questionnaire.

## Discussion

This study is the first systematic review and meta‐analysis that comprehensively investigated the interrelationship between sarcopenia and multiple neurocognitive disorders. Different from previous meta‐analyses, we expanded the scope to a broad spectrum of cognitive dysfunction, including MCI, AD and other forms of dementia.

We have found a positive association between sarcopenia and different neurocognitive disorders. More specifically, sarcopenia increased the odds of having MCI, AD and non‐AD dementia by 58%, 197% and 68%, respectively. However, our data also revealed that the magnitude of the associations may differ across study populations, sarcopenia definition, clinical stage of cognitive impairment and the tools used for evaluating cognitive function.

Our meta‐analysis showed that the odds of having MCI in the sarcopenia group was 1.58 times higher than that in the non‐sarcopenia group. Likewise, results from previous cross‐sectional and cohort studies have consistently shown that sarcopenia is associated with MCI. However, a somewhat heterogeneous magnitude of associations was found. For instance, findings from the subgroup analyses demonstrated that the statistical significance of the associations differed across used tool to define or diagnose MCI. These heterogeneous results may potentially be explained due to the lack of using validated criteria to diagnose MCI. For instance, only 5 of the 14 included studies in this meta‐analysis used validated criteria to diagnose MCI (diagnosis by neurologists, NIA‐AA criteria or the modified Petersen criteria).

The majority of studies used the MMSE test and MoCA test to assess MCI, both in the community and in the hospitalized setting. These studies showed that the association between sarcopenia and MMSE‐defined MCI was higher than the association of sarcopenia with MoCA‐defined MCI. Thus, the use of these two different screening tools may lead to different estimates of the association between sarcopenia and MCI. However, it is essential to note that these two screening tests are not designed to make a definitive diagnosis of MCI or differentiate with the more severe form of cognitive impairment, dementia.^93^ Indeed, no clear classification of cut‐off values to define MCI has been developed yet for these tests, and studies have not taken into account the impact of the level of severity of cognitive impairment on the found results. Therefore, it may be possible that the studies using these tests included not just individuals with MCI but also individuals who may have a moderate or severe form of cognitive impairment or even dementia.

Thus, the use of a clinical evaluation, including additional cognitive assessments and consideration of validated criteria such as the NIA‐AA criteria or (modified) Petersen criteria, is necessary to properly distinguish between cognitive impairment, MCI and dementia.^93^

A vast amount of studies have investigated the association between sarcopenia and MCI. Nevertheless, this relationship is still not fully understood and may even be controversial. For instance, the study by Yuenyongchaiwat and Boonsinsukh is the only study included in this systematic review to find a negative association between sarcopenia and MCI, indicating that individuals with sarcopenia were less likely to have MCI.^90^ This unusual finding may be explained by the lack of correct adjustment for important confounders. However, additional research is needed to explain the underlying factors that may explain this complex relationship between sarcopenia and MCI.

The association between sarcopenia and dementia, including various forms of dementia such as AD and Parkinson's dementia, has also been investigated in previous studies, although the literature on this specific association is not as extensive compared to the association between sarcopenia and MCI. When looking into the studies exploring the different types of dementia, our findings are indicative of an association between sarcopenia and AD (OR = 2.97, 95% CI 2.15–4.08), regardless of the population characteristics or used tool to diagnose sarcopenia or AD.

It is important to note that the significance and magnitude of the association between sarcopenia and dementia may differ depending on the study population. There was a stronger association between sarcopenia and dementia (both AD and non‐AD) in the hospitalized setting than in the community‐dwelling setting. This may be explained by the following: Hospitalization may lead to functional impairment and sarcopenia due to prolonged immobilization and bed rest and may also contribute to delirium or acute cognitive changes, which can influence the diagnosis of cognitive impairment in this setting.^94^, ^95^ In addition, hospitalized settings may use different diagnostic criteria or assessment tools for both sarcopenia and MCI/dementia compared to the community‐dwelling setting, which can influence the observed prevalence rates and association between both conditions.^94^ Lastly, selection bias may also play a role in the high prevalence of sarcopenia and MCI/dementia in the hospitalized setting. For instance, individuals in the hospitalized setting are often individuals with more severe illnesses and higher levels of medical complexity, leading to a different prevalence and strength or magnitude of association between sarcopenia and cognitive impairment. On the other hand, community‐dwelling older adults may have a wide range of health and functional abilities that may lead to more variability in the association between sarcopenia and cognitive dysfunction. More high‐quality research is needed to better understand the relationship between sarcopenia and cognitive impairment, including MCI and dementia, in both hospitalized and community‐dwelling settings.

Our results of the association between sarcopenia and other forms of (non‐AD) dementia are less clear. A possible explanation for the discrepancies in these results may be the lack of specifics on the dementia subtype and the absence of the use of validated criteria to diagnose non‐AD dementia. For instance, only four of the nine included studies in this meta‐analysis used validated criteria to diagnose dementia, of which only two studies specified the dementia subtype.

Several studies that have reported on non‐AD dementia used inadequate diagnostic tools to define dementia (e.g., MoCA and MMSE). The big drawback of the use of these tests is that they are unable to accurately differentiate the severity of cognitive dysfunction, which can lead to misclassification errors.^96^ For instance, individuals with MCI or early stage AD can be erroneously included in studies focusing on non‐AD dementia. Therefore, this lack of precision in the included studies presented challenges in interpretation, rendering it impossible to draw any definite conclusions on the association of sarcopenia and cognitive dysfunction, including MCI and dementia subtypes. Moreover, given the limited number of studies exploring the relationship between sarcopenia and dementia (both AD and non‐AD dementia) included in our meta‐analysis (12 studies), we cannot draw strong conclusions on the relationship between sarcopenia and dementia (both AD and non‐AD).

There are several potential mechanisms that may explain the association between sarcopenia and MCI or dementia (both AD and non‐AD). For instance, age, gender, decreased physical activity and education level are factors that may be influencing both conditions.^13^ All the included studies in our meta‐analysis have adjusted their analyses for several putative confounders. Even though the used putative covariates might have differed between the studies, most studies included the variables age, sex, education, physical activity (measured by questionnaires) and common comorbidities as confounders in their analyses. Some of the studies that did not find a significant association between sarcopenia and cognitive dysfunction have additionally adjusted for nutritional status, quality of life, performance on ADLs or interleukin‐6 (IL‐6).^47^, ^62^, ^89^ This observation suggests that the relationship between sarcopenia and cognitive decline may not be simply explained by solely the ageing process, physical activity level or comorbidities. The commonly accepted hypothesis for the association between sarcopenia and cognitive dysfunction is the shared underlying pathophysiological mechanisms. Both sarcopenia and cognitive dysfunction are age‐related conditions influenced by similar biological processes, including chronic inflammation, oxidative stress, hormonal changes and metabolic abnormalities.^13^

One of the major strengths of this systematic review and meta‐analysis is that it extensively summarized the interrelationship between sarcopenia and cognitive dysfunction, including MCI, AD and other forms of dementia. Similar results were found in previous meta‐analyses exploring the relationship between sarcopenia and (mild) cognitive impairment. However, unlike prior meta‐analyses, we solely focused on studies reporting on MCI and excluded studies that did not specify the severity of the cognitive impairment. Studies reporting on cognitive impairment may be mixed with individuals with also moderate or severe cognitive impairment, rather than MCI as a specific clinical syndrome, demonstrating a different strength of association with sarcopenia. In addition, we broadened the scope of this meta‐analysis to include a range of various forms of dementia, both AD dementia and non‐AD dementia. Lastly, since the rapid growth of studies published in this area of study, more recent (longitudinal) studies were included in this up‐to‐date meta‐analysis.

Several limitations should be considered when interpreting our findings. First, the majority of the included studies in our analysis had a cross‐sectional study design, which precludes definitive conclusions regarding the causal relationship between sarcopenia and cognitive dysfunction. While our study provides valuable evidence supporting the association between sarcopenia and cognitive dysfunction, the directionality and causality of this relationship are yet to be fully elucidated. From the four included longitudinal cohort studies exploring this relationship, three studies reported that sarcopenia was significantly associated with developing MCI or AD after follow‐up (with a follow‐up range of 3–8 years).^11^, ^40^, ^65^ Results from these studies shed light on whether sarcopenia precedes cognitive decline. Yet a bidirectional and interrelated relationship between sarcopenia and cognitive decline may also be possible.^89^ It is important to note that most longitudinal studies did not assess the correlation from the other direction, that is, whether having cognitive dysfunction could predict the risk of having sarcopenia. However, one study found that having probable dementia at baseline was significantly associated with incident sarcopenia after 2 years of follow‐up, but not after 4 years of follow‐up. Also, in this study, some individuals with initial sarcopenia had reverted to having no sarcopenia.^89^ This finding suggests a dynamic and complex relationship between sarcopenia and cognitive dysfunction with each condition influencing and exacerbating the other over time. More studies are needed to provide insights into whether sarcopenia precedes the onset of cognitive dysfunction or vice versa, in order to better understand the cause‐and‐effect nature of this association. Moreover, only two interventional studies were included in this systematic review that explored the impact of interventions, such as exercise programmes, on both sarcopenia and cognitive impairment.^38^, ^91^ Large‐scale prospective interventional studies are needed to further evaluate whether addressing sarcopenia through interventions can have positive effects on cognitive function. In addition, future longitudinal studies should also include relevant biomarkers to explore the mechanisms involved in both conditions. Secondly, the heterogeneity in the used definition of sarcopenia and tool to diagnose cognitive status may introduce variability and may influence the strength of the observed association. More specifically, consensus on standardized scales to measure sarcopenia or cognitive dysfunction would enhance comparability between studies. For instance, a strong current challenge in the field of sarcopenia is the lack of a universally accepted established definition, which directly impacts the prevalence rate of sarcopenia and its specific association with health outcomes, including cognitive dysfunction.^97^ Moreover, the heterogeneity in cut‐off values, using various different methods to measure muscle mass (e.g., dual‐energy X‐ray absorptiometry and bioelectrical impedance analysis), and the inappropriate use of screening tools (e.g., SARC‐F) to diagnose sarcopenia may also have a huge impact on the association of sarcopenia with cognitive dysfunction. Not only the lack of consensus on the criteria and cut‐off points for evaluating sarcopenia has yet to be established, but the diversity of measurement methods to diagnose MCI, AD and other forms of dementia is also a big limitation in the research field. This heterogeneity and lack of specific dementia diagnostic tools have made it very difficult to compare the included studies. Future research with a larger number of studies using validated diagnostic criteria is necessary to further confirm and deepen our understanding of the association between sarcopenia and MCI or dementia, including its various types.

Thirdly, we also acknowledge the fact that the majority of the included studies were performed in Asian and European populations, resulting in a population bias and therefore limiting the representativeness of the general population. Fourthly, we cannot rule out the possibility that excluding those studies that reported on unspecified impairment may have included individuals with MCI or dementia (both AD and non‐AD). A fifth limitation is that most studies did not meet high‐quality criteria, and potential publication bias could not be ruled out. However, based on the sensitivity analyses, these studies did not negatively affect the results. Lastly, we did not take into account the dose–response relationship between sarcopenia and cognitive disorders. For instance, our results showed that there is a significant association between sarcopenia and cognitive disorders, which may be of higher magnitude in the context of a greater degree of sarcopenia and/or a clinical stage of cognitive impairment. For instance, the study by Someya et al. demonstrated that sarcopenia was significantly associated with non‐AD dementia but not with MCI.^67^ Thus, this dose–response relationship between sarcopenia and cognitive dysfunction may modify the association between both conditions. Future research should stratify and investigate the influence of different stages of sarcopenia on different clinical stages of cognitive impairment.

Although this meta‐analysis gives an extensive overview of the existing evidence on the interrelationship between sarcopenia and cognitive dysfunction, the limitations present in the included studies urge cautious interpretation of our findings and warrant more high‐quality studies. Future research should therefore focus on more longitudinal studies to explore the cause–nature relationship between sarcopenia and cognitive impairment, using well‐defined criteria to diagnose both sarcopenia and the cognitive outcomes.

## Conclusions

Our analysis supports the association between sarcopenia and cognitive decline, including MCI, AD and other forms of dementia. This emerging evidence suggesting a connection between sarcopenia and cognitive dysfunction adds another layer to the detrimental consequences of sarcopenia on overall health and well‐being. Future research should focus on high‐quality longitudinal investigations to determine causality and explore potential therapeutic strategies aimed at preserving muscle mass, strength and cognitive function in the ageing population.

## Conflict of interest statement

All authors declare that they have no conflicts of interest related to this manuscript.

## Supporting information


**Table S1.** Overview search strategy in databases
**Table S2.** Summary table of characteristics included studies (qualitative synthesis)
**Table S3a.** Quality assessment of cross‐sectional studies using the modified Newcastle‐Ottawa scale
**Table S3b.** Quality assessment of cohort studies using the Newcastle‐Ottawa scale
**Table S3c.** Quality assessment of interventional studies
**Table S4.** Sensitivity analyses
**Figure S1.** Funnel plot for publication bias. (a) Correlation between mild cognitive impairment and sarcopenia. (b) Correlation between Alzheimer's disease and sarcopenia. (c) Correlation between non‐AD dementia and sarcopenia
**Table S5.** Egger's test for publication bias
