# Supplementary material for: Meta‐analysis on the interrelationship between sarcopenia and mild cognitive impairment, Alzheimer's disease and other forms of dementia
Source: J Cachexia Sarcopenia Muscle. 2024 May 7;15(4):1240–53. doi: 10.1002/jcsm.13485 (PMC11294028; doi:10.1002/jcsm.13485)
Supplement: Supplementary file 1 — Table S1. Overview search strategy in databases Table S2. Summary table of characteristics included studies (qualitative synthesis) Table S3a. Quality assessment of cross‐sectional studies using the modified Newcastle‐Ottawa scale Table S3b. Quality assessment of cohort studies using the Newcastle‐Ottawa scale Table S3c. Quality assessment of interventional studies Table S4. Sensitivity analyses Figure S1. Funnel plot for publication bias. (a) Correlation between mild cognitive impairment and sarcopenia. (b) Correlation between Alzheimer's disease and sarcopenia. (c) Correlation between non‐AD dementia and sarcopenia Table S5. Egger's test for publication bias [file JCSM-15-1240-s001.docx]

**Supplementary Material 1: Overview search strategy in databases**

| **PUBMED** | "Muscular Atrophy"[Mesh] OR “sarcopenia”[tiab] OR “sarcopenic”[tiab] OR “Muscle Weakness”[Mesh] OR “Muscle Strength”[Mesh] OR “Physical Functional Performance”[Mesh] OR “functional performan*”[tiab] OR “physical performan*”[tiab] OR “anabolic resistan*”[tiab] OR “muscle aging*”[tiab] OR “muscular aging*”[tiab] OR “muscle ageing*”[tiab] OR “muscular ageing*”[tiab] OR “muscle cell degeneration*”[tiab] OR “muscle degeneration*”[tiab] OR “muscular degeneration*”[tiab] OR “muscle fiber degeneration*”[tiab] OR “muscle fibre degeneration*”[tiab] OR “muscle recession*”[tiab] OR “muscular recession*”[tiab] OR “muscle wasting*”[tiab] OR “myoatroph*”[tiab] OR “myodegeneration*”[tiab] OR “myofibrillar degeneration*”[tiab] OR “muscle insufficien*”[tiab] OR “muscular insufficien*”[tiab] OR “muscle volume*”[tiab] OR “muscular volume*”[tiab] OR “muscle force*”[tiab] OR “muscle power*”[tiab] OR “muscular force*”[tiab] OR “muscular power*”[tiab] OR ((musc*[tiab]) AND (strength[tiab] OR weak*[tiab] OR atroph*[tiab] OR mass[tiab] OR qualit*[tiab]))  **AND**  "Dementia"[Mesh:NoExp] OR “[Dementia, Vascular](https://www.ncbi.nlm.nih.gov/mesh/68015140)”[Mesh] OR “Frontotemporal Dementia”[Mesh] OR “dement*”[tiab] OR “amentia*”[tiab] OR “Alzheimer Disease”[Mesh] OR “Alzheimer*”[tiab] OR "mild cognitive impairment*"[tiab] |
| --- | --- |
| **EMBASE** | 'muscle atrophy'/exp OR ‘sarcopenia*’:ti,ab,kw OR ‘sarcopenic’:ti,ab,kw OR 'muscle weakness'/de OR ‘arm weakness’/exp OR ‘limb weakness’/exp OR ‘Muscle Strength’/exp OR ‘physical performance'/de OR ‘functional performan*’:ti,ab,kw OR ‘physical performan*’:ti,ab,kw OR 'anabolic resistance'/exp OR ‘anabolic resistan*’:ti,ab,kw OR ‘muscle mass’/exp OR ‘myoatroph*’:ti,ab,kw OR ‘myodegeneration*’:ti,ab,kw OR ‘myofibrillar degeneration*’:ti,ab,kw OR ‘muscle insufficien*’:ti,ab,kw OR ‘muscular insufficien*’:ti,ab,kw OR ‘muscle volume*’:ti,ab,kw OR ‘muscular volume*’:ti,ab,kw OR ((musc*) NEAR/3 (strength OR weak* OR atroph* OR mass OR qualit* OR aging OR ageing)):ti,ab,kw OR ((musc*) NEAR/3 (degeneration OR recession OR wasting OR force OR power)):ti,ab,kw NOT (‘conference abstract’/it)  **AND**  'dementia'/de OR 'dement*':ti,ab,kw OR 'frontotemporal dementia'/exp OR 'senile dementia'/exp OR 'amentia*':ti,ab,kw OR 'Alzheimer disease'/exp OR 'Alzheimer*':ti,ab,kw OR ‘mild cognitive impairment'/exp OR 'mild cognitive impairment*’:ti,ab,kw NOT (‘conference abstract’/it) |
| **Web of Science** | TS=(“sarcopenia” OR “sarcopenic” OR “anabolic resistan*” OR “functional performan*” OR “physical performan*” OR “myoatroph*” OR “myodegeneration*” OR “myofibrillar degeneration*” OR “muscle insufficien*” OR “muscular insufficien*” OR “muscle volume*” OR “muscular volume*” OR (“musc*” NEAR/3 (“strength” OR “weak*” OR “atroph*” OR “mass” OR “qualit*” OR “aging” OR “ageing”)) OR ((“musc*”) NEAR/3 (“degeneration” OR “recession” OR “wasting” OR “force” OR “power”)))  NOT DT=(“meeting abstract”)  **AND**  TS=(“dement*” OR “amentia*” OR “Alzheimer*” OR "mild cognitive impairment*")  NOT DT=(“meeting abstract”) |
| **Cochrane Library** | ([mh “Muscular Atrophy”] OR [mh “Muscle Weakness”] OR [mh “Muscle Strength”] OR [mh “Physical Functional Performance”]) OR sarcopenia* OR sarcopenic OR (functional NEXT performan*) OR (physical NEXT performan*) OR (anabolic NEXT resistan*) OR myoatroph* OR myodegeneration* OR (myofibrillar NEXT degeneration*) OR (muscle NEXT insufficien*) OR (muscular NEXT insufficien*) OR (muscle NEXT volume*) OR (muscular NEXT volume*) OR (musc* NEAR/3 (strength OR weak* OR atroph* OR mass OR qualit* OR aging OR ageing)):ti,ab,kw OR (musc* NEAR/3 (degeneration OR recession OR wasting OR force OR power)):ti,ab,kw  **AND**  ([mh ^“Dementia”] OR [mh “Alzheimer Disease”] OR [mh “[Dementia, Vascular](https://www.ncbi.nlm.nih.gov/mesh/68015140)”] OR [mh “Frontotemporal Dementia”]) OR (dement* OR amentia* OR Alzheimer* OR (“mild cognitive” NEXT impairment*)):ti,ab,kw |
| **CINAHL** | ((MH "Muscular Atrophy+") OR (MH "Muscle Weakness+") OR (MH "Muscle Strength+") OR (MH "Physical Performance")) OR TI ( “sarcopenia” OR “sarcopenic” OR “functional performan*” OR “physical performan*” OR “anabolic resistan*” OR “myoatroph*” OR “myodegeneration*” OR “myofibrillar degeneration*” OR “muscle insufficien*” OR “muscular insufficien*” OR “muscle volume*” OR “muscular volume*” OR (musc* N3 (strength OR weak* OR atroph* OR mass OR qualit* OR aging OR ageing)) OR (musc* N3 (degeneration OR recession OR wasting OR force OR power))) OR AB ( “sarcopenia” OR “sarcopenic” OR “functional performan*” OR “physical performan*” OR “anabolic resistan*” OR “myoatroph*” OR “myodegeneration*” OR “myofibrillar degeneration*” OR “muscle insufficien*” OR “muscular insufficien*” OR “muscle volume*” OR “muscular volume*” OR (musc* N3 (strength OR weak* OR atroph* OR mass OR qualit* OR aging OR ageing)) OR (musc* N3 (degeneration OR recession OR wasting OR force OR power)))  **AND**  ((MH "Dementia") OR (MH "Dementia, Vascular+") OR (MH "Dementia, Senile") OR (MH "Alzheimer's Disease") OR (MH "Mild Cognitive Impairment")) OR TI (“dement*” OR “amentia*” OR “Alzheimer*” OR "mild cognitive impairment*") OR AB (“dement*” OR “amentia*” OR “Alzheimer*” OR "mild cognitive impairment*") |
| **SCOPUS** | TITLE-ABS( “sarcopenia” OR “sarcopenic” OR “functional performan*” OR “physical performan*” OR “anabolic resistan*” OR “myoatroph*” OR “myodegeneration*” OR “myofibrillar degeneration*” OR “muscle insufficien*” OR “muscular insufficien*” OR “muscle volume*” OR “muscular volume*” OR (“musc*” W/3 (“strength” OR “weak*” OR “atroph*” OR “mass” OR “qualit*” OR “aging” OR “ageing”)) OR (“musc*” W/3 ("degeneration” OR “recession” OR “wasting” OR “force” OR “power”))) OR AUTHKEY( “sarcopenia” OR “sarcopenic” OR “functional performan*” OR “physical performan*” OR “anabolic resistan*” OR “myoatroph*” OR “myodegeneration*” OR “myofibrillar degeneration*” OR “muscle insufficien*” OR “muscular insufficien*” OR “muscle volume*” OR “muscular volume*” OR (“musc*” W/3 (“strength” OR “weak*” OR “atroph*” OR “mass” OR “qualit*” OR “aging” OR “ageing”)) OR (“musc*” W/3 ("degeneration” OR “recession” OR “wasting” OR “force” OR “power”)))  **AND**  TITLE-ABS(“dement*” OR “amentia*” OR “Alzheimer*” OR "mild cognitive impairment*") OR AUTHKEY(“dement*” OR “amentia*” OR “Alzheimer*” OR "mild cognitive impairment*") |
| **Sportdiscus** | DE "MUSCULAR atrophy" OR DE "MUSCLE strength" OR DE “[MUSCLE aging](javascript:XslPostBack('ctl00$ctl00$MainContentArea$MainContentArea$xslResults','ThesaurusLink','LinkTarget%7CauthorityList%24LinkTerm%7CDE%2B%2522MUSCLE%2Baging%2522');)” OR DE “MUSCLE mass” OR **TI** (“sarcopenia” OR “sarcopenic” OR “anabolic resistan*” OR “functional performan*” OR “physical performan*” OR “myoatroph*” OR “myodegeneration*” OR “myofibrillar degeneration*” OR “muscle insufficien*” OR “muscular insufficien*” OR “muscle volume*” OR “muscular volume*” OR (“musc*” W3 (“strength” OR “weak*” OR “atroph*” OR “mass” OR “qualit*” OR “aging” OR “ageing”)) OR ((“musc*”) W3 (“degeneration” OR “recession” OR “wasting” OR “force” OR “power”))) OR AB (“sarcopenia” OR “sarcopenic” OR “anabolic resistan*” OR “functional performan*” OR “physical performan*” OR “myoatroph*” OR “myodegeneration*” OR “myofibrillar degeneration*” OR “muscle insufficien*” OR “muscular insufficien*” OR “muscle volume*” OR “muscular volume*” OR (“musc*” W3 (“strength” OR “weak*” OR “atroph*” OR “mass” OR “qualit*” OR “aging” OR “ageing”)) OR ((“musc*”) W3 (“degeneration” OR “recession” OR “wasting” OR “force” OR “power”))) OR KW (“sarcopenia” OR “sarcopenic” OR “anabolic resistan*” OR “functional performan*” OR “physical performan*” OR “myoatroph*” OR “myodegeneration*” OR “myofibrillar degeneration*” OR “muscle insufficien*” OR “muscular insufficien*” OR “muscle volume*” OR “muscular volume*” OR (“musc*” W3 (“strength” OR “weak*” OR “atroph*” OR “mass” OR “qualit*” OR “aging” OR “ageing”)) OR ((“musc*”) W3 (“degeneration” OR “recession” OR “wasting” OR “force” OR “power”)))  **AND**  DE “DEMENTIA” OR DE “[ALZHEIMER'S disease](javascript:XslPostBack('ctl00$ctl00$MainContentArea$MainContentArea$xslResults','ThesaurusLink','LinkTarget%7CauthorityList%24LinkTerm%7CDE%2B%2522ALZHEIMER%5C%27S%2Bdisease%2522');)” OR TI (“dement*” OR “amentia*” OR “Alzheimer*” OR "mild cognitive impairment*") OR AB (“dement*” OR “amentia*” OR “Alzheimer*” OR "mild cognitive impairment*") OR KW (“dement*” OR “amentia*” OR “Alzheimer*” OR "mild cognitive impairment*") |
| **PEDro** | Abstract and title: Alzheimer, mild cognitive impairment or dementia; Problem: muscle weakness |
| **CLINICALTRIAL** | Condition or disease: Sarcopenia; Study type: interventional studies; Study results: studies with results; Status: completed; Eligibility: Adult (18-64), Older adult (65+) |

**Supplementary Material 2: Summary table of characteristics included studies (qualitative synthesis)**

| 1. Results from studies investigating the association between mild cognitive impairment and sarcopenia | | | | | | |
| --- | --- | --- | --- | --- | --- | --- |
| Cross-sectional studies | | | | | | |
| Author, year | **Country** | **Population** | **Sample size** | **Assessment of cognition** | **Assesment of sarcopenia** | **Main results** |
| Bai, 2021^19^ | China | Community-dwelling older adults (>80 years)  Mean age: 86.3  % female subjects: 71.0% | 428 | Modified Petersen criteria | AWGS2 | The prevalence of MCI was significantly different dependent on sarcopenia status: The prevalence of MCI was 28.9% in sarcopenic older adults and 17.4% in non-sarcopenic older adults (p<0.005). Sarcopenia was significantly associated with MCI (OR:1.86 95% CI:1.04–3.33). |
| Chen, 2021^27^ | China | Community-dwelling older adults (≥60 years)  Mean age: 71.9  % female subjects:   - MCI group: 69.8% - Normal cognition group: 56.6% | 1394 | Modified Petersen criteria | AWGS1 | The prevalence of sarcopenia was significantly different dependent on cognitive status: the prevalence of sarcopenia was 8.9% in subjects with normal cognitive status and 18.7% in subjects with MCI (p<0,001). Sarcopenia was significantly associated with MCI (OR: 1.67 95% CI 1.04-2.68) |
| Chong, 2015^31^ | Singapore | Community-dwelling older adults attending the memory clinic with MCI or mild and moderate AD (≥50 years)  Mean age: 70.8  % female subjects: 67.2% | 299 | Modified Petersen criteria | AWGS1 | The prevalence of sarcopenia was significantly different dependent on cognitive status (p=0,000): 24.6% in healthy controls, 37.5% in MCI, 47.1% in mild AD and 60.0% in moderate AD. |
| Ida, 2017^42^ | Japan | Community-dwelling older adults with diabetes  (≥65 years)  Mean age: 71.8  % female subjects: 40.0% | 250 | TYM (Japanese version) | SARC-F | The prevalence of MCI was significantly different dependent on sarcopenia status: the prevalence of MCI was 69.3% in subjects with sarcopenia vs 36.6% in subjects without sarcopenia (p<0,001). Sarcopenia was significantly associated with MCI (OR: 2.96 95% CI:1.09-7.70) |
| Iritani, 2021^43^ | Japan | Community-dwelling older adults  Mean age: 79.5  % female subjects: 65.1% | 135 | MMSE | AWGS1 | The prevalence of sarcopenia was significantly different according to cognitive status (p<0,01): 25% in subjects with normal cognition; 65.2% in subjects with MCI and 45.8% in subjects with AD. |
| Jacob, 2021^44^ | 6 low-and middle-income countries; China, Ghana, India, Mexico, Russia, South Africa | Community-dwelling older adults (≥65 years)  Mean age: 72.2  % female subjects: 54.8% | 12912 | NIA-AA | EWGSOP2 | The prevalence of MCI was significantly different dependent on sarcopenia status: the prevalence of MCI was 27.6% in subjects with sarcopenia vs 16.8% in subjects without sarcopenia. Sarcopenia was significantly associated with MCI in all countries, with the exception of South Africa (Overall OR: 1.60 95% CI:1.32-1.93, with a low level of between-country heterogeneity (I^2^=0.0%)). |
| Kim, 2014^46^ | Korea | Older adults with end-stage renal disease (≥50 years)  Mean age: 63.9  % female subjects: 43.2% | 95 | MMSE | EWGSOP1 | Sarcopenia was significantly associated with MCI (OR: 6.35 95%CI:1.62-34.96) |
| Kimura,2018^47^ | Japan | Community-dwelling older adults with MCI and early-stage AD (65-89years)  Mean age: 77.2  % female subjects: 63.4% | 205 | NIA-AA | AWGS1 | The prevalence of sarcopenia was 14.6% in patients with MCI or early-stage AD. |
| Lee, 2018^52^ | Korea | Community-dwelling older women (≥65 years)  Mean age: 74.3  % female subjects: 100% | 201 | MMSE | AWGS1 | Pre-sarcopenia was significantly associated with MCI (OR:2.36 95% CI: 0.91-6.00), whereas sarcopenia was not significantly associated with MCI (OR: 4.50 95% CI 1.31-15.32). |
| Liu, 2020^56^ | China | Community-dwelling multi-ethnic older adults  (≥50 years)  Mean age: 62.4  % female subjects: 63.8% | 4500 | SPMSQ | AWGS1 | The prevalence of CI was significantly different dependent on sarcopenia status: the prevalence of MCI was 14.0% in subjects with sarcopenia vs 9.7% in subjects without sarcopenia and the prevalence of moderate-severe CI was 8.3% in subjects with sarcopenia vs 2.2% in subjects without sarcopenia (p<0,001). CI significantly associated with sarcopenia with a dosage effect (mild cognitive impairment: OR:1.41, 95% CI 1.10–1.82; moderate/severe cognitive impairment: OR: 3.05, 95%CI 2.08–4.49). After gender stratification, the association between mild cognitive impairment with sarcopenia in male adults was not significant, while still significant in female adults. Moderate/severe cognitive impairment was significantly associated with sarcopenia in both male and female. |
| Machii, 2020^57^ | Japan | Older adults with type 2 diabetes mellitus  Mean age: 69  % female subjects: 44% | 438 | MoCA (Japanese version) | AWGS2 | The prevalence of sarcopenia was significantly different according to cognitive status: the prevalence of sarcopenia was 13% in subjects with MCI vs 4% in subjects without MCI (p<0,01). Sarcopenia was not significantly associated with MCI (OR: 1.39 95% CI: 0.59-3.28). |
| Oba, 2022^59^ | Japan | Community-dwelling older adults with cardiometabolic diseases without severe CI  Mean age: 78.3  % female subjects: 65.5% | 417 | MoCA (Japanese version) | AWGS2 | Sarcopenia was not significantly associated with MCI (OR not mentioned). |
| Ogama, 2019^60^ | Japan | Community-dwelling adults with type 2 diabetes mellitus (≥65years)  Mean age: 75  % female subjects: 48.8% | 69 | NIA-AA and Petersen criteria | AWGS1 | The prevalence of sarcopenia was significantly different dependent on cognitive status (p=0.021): 21.9% in subjects with MCI and 2.7% in subjects with normal cognition. |
| Papachristou, 2015^63^ | Great-Britain | Community-dwelling older British men (71-92 years)  Mean age: 78.09  % female subjects: 0% | 1570 | TYM | EWGSOP1 & FNIH | The prevalence of sarcopenia was significantly different dependent on cognitive status: the prevalence of severe sarcopenia (EWGSOP1) was 2.8% in subjects with MCI vs 1.5% in subjects with normal cognitive status vs 7.5% in subjects with severe CI (p<0,001). In addition, the prevalence of sarcopenia (FNIH) was 3.2% in subjects with MCI vs 1.8% in subjects with normal cognitive status vs 5.7% in subjects with severe CI (p<0,04). 1) EWGSOP1-definition: Severe sarcopenia was not significantly associated with MCI (OR:1.40 95% CI:0.56-3.51) or severe CI (OR=2.84 95% CI:0.89-9.09). 2) FNIH-definition: Sarcopenia was not significantly associated with MCI (OR: 1.72 95% CI 0.67-4.40) and severe CI (OR:1.47 95% CI 0.35-6.12). |
| Someya, 2022^67^ | Japan | Community-dwelling older adults (65-84 years old)  Mean age: 73.1  % female subjects: 57.6% | 1615 | MoCA | AWGS2 | Sarcopenia was not significantly associated with MCI (OR: 1.33 95% CI:0.92-1.93). |
| Sugimoto, 2022^71^ | Japan | Community-dwelling older adults and clinically diagnosed with MCI or AD (65-89 years)  Mean age: 77.7  % female subjects: 63.8% | 1181 | NIA-AA | Modified EWGSOP2 diagnostic flow: Asian-gender specific cut-off values for sarcopenia -defining parameters | The prevalence of sarcopenia was higher in subjects with AD than in subjects with MCI. AD: 30.2% (men) and 23.8% (women); MCI: 17.5% (men) and 12.7% (women). |
| Sugimoto, 2016^72^ | Japan | Community-dwelling older adults attending the Memory Clinic (≥60years)  Mean age: 77.3  % female subjects: 66.8 | 418 | Petersen criteria | EWGSOP1 | The clinical stage of cognitive impairment was significantly different in sarcopenic older adults (p=0,047). The prevalence of normal cognition, MCI and AD in subjects with sarcopenia was 8.6%, 12.5% and 23.3%, respectively, whereas the prevalence of normal cognition, MCI and AD in subjects without sarcopenia was 91.4%, 87.5% and 76.7%, respectively. |
| Tay, 2018^77^ | Singapore | Community-dwelling older adults with MCI and mild to moderate AD (>50years)  Mean age: 76.7  % female subjects: 64.8% | 108 | Modified Petersen criteria | Modified EWGSOP1: Asian-gender specific cut-off values for sarcopenia -defining parameters | The prevalence of sarcopenia was not significantly different dependent on cognitive status (p=0,471): In subjects with MCI: 11.3% had sarcopenia and 28.6% had pre-sarcopenia. In subjects with mild AD: 64.3% had sarcopenia and 69.8% had pre-sarcopenia. In subjects with moderate AD: 18.9% had sarcopenia and 7.1% had presarcopenia. |
| Li, 2022^53^ | China | Hospitalized male patients (≥65years)  Mean age: 79.6  % female subjects: 0% | 250 | MMSE | AWGS2 | The prevalence of sarcopenia was significantly different dependent on cognitive status (p<0,001): 14.9% in subjects with normal cognitive status, 39.0% in subjects with MCI and 75.0% in subjects with moderate CI. |
| Umegaki, 2020^81^ | Japan | Community-dwelling older adults with early dementia and MCI  Mean age: 80.1  % female subjects: 54.1% | 74 | MMSE | AWGS2 | The prevalence of sarcopenia was 47% in patients with MCI and early dementia. |
| Woo, 2015^84^ | China | Community-dwelling older adults (≥65years)  58.9% of the subjects were >76 years old  % female subjects: 85.4% | 816 | Abbreviated Memory Inventory for the Chinese | SARC-F | 63.7% of the subjects had both sarcopenia and MCI. |
| Yamada, 2021^86^ | Japan | Community-dwelling older adults at the Memory Clinic (≥65years)  Mean age: 77.5  % female subjects: 62.5% | 1774 | Petersen criteria | Modified EWGSOP2: Asian-gender specific cut-off values for sarcopenia -defining parameters | The prevalence rates of both MCI (10.5%) and dementia (84.1%%) were significantly higher in the sarcopenia group than in the non-sarcopenia group (MCI: 21% and dementia: 64.5%) (p<0,001). |
| Yuenyongchaiwat, 2020^91^ | Thailand | Community-dwelling older adults (≥60years)  Mean age: 66.1  % female subjects: 67.3% | 330 | MoCA (Thailand) | AWGS1 | Sarcopenia was significantly associated with MCI (OR: 0.215 95% CI 0.047-0.987). |
| Hu, 2021^40^ | China | Community-dwelling older adults (≥50years)  Mean age: 61.9  % female subjects: 63.6% | 3147 | SPSMQ | AWGS2 | The prevalence of CI was significantly different dependent on sarcopenia status: the prevalence of MCI was 11.6% in subjects with sarcopenia vs 15.9% in subjects with severe sarcopenia vs 9.4 % in subjects without sarcopenia; the prevalence of moderate-severe CI was 3.1% in subjects with sarcopenia vs 12.3% in subjects with severe sarcopenia vs 2.0% in subjects without sarcopenia (p<0,001). Sarcopenia was significantly associated with cognitive impairment (ß= 0.208, 95% CI 0.072 to 0.344). |
| Rodrigues-Rejon, 2020^65^ | Spain | Long-term care home residents (≥70years)  Mean age: 84.9  % female subjects: 75% | 249 | Pfeiffer test | EWGSOP2 | The prevalence of CI was significantly different dependent on sarcopenia status: the prevalence of MCI was 71% in subjects with sarcopenia vs 29% in subjects without sarcopenia; the prevalence of moderate CI is 68% in subjects with sarcopenia vs 32% in subjects without sarcopenia; the prevalence of severe CI is 71% in subjects with sarcopenia vs 29% in subjects without sarcopenia (p=0,004). Moderate or severe CI was significantly associated with having sarcopenia (OR: 2.40; 95% CI 0.85-6.39) |
| Demura, 2023^34^ | Japan | Community-dwelling older adults with normal cognition to AD  Mean age: 80.90  % female subjects: 57.90% | 95 | MMSE | AWGS2 | The prevalence of sarcopenia was significantly different dependent on cognitive status (p<0,05): 37.9% (normal cognition); 73.9% (MCI) and 62.8% (AD). |
| O'Donovan, 2022^58^ | Colombia | Community-dwelling older adults (≥60years)  Mean age: 71  % female subjects: 59.64% | 5760 | Short version of the MMSE (Total score 19) | EWGSOP2 | The prevalence of MCI was significantly different dependent on sarcopenia status: the prevalence of MCI was 22% in subjects with sarcopenia vs 8% in subjects without sarcopenia. Sarcopenia was only significantly associated with MCI in subjects with normal BMI (OR: 1.84 95% CI 1.25-2.71) or obese subjects (OR:1.62 95% CI 1.07-2.48). |
| Wang, 2022^83^ | China | Community-dwelling older adults (65-85years)  Mean age: 70  % female subjects: 59.9% | 1050 | MoCA with education-adjusted cut-offs and clinical diagnosis by neurologist | AWGS2 | 7.52% of the subjects had both MCI and sarcopenia. |
| Lee, 2023^50^ | South-Korea | Community-dwelling older adults (≥60years)  Mean age: 74.0  % female subjects: 40.2% | 286 | Diagnosed by neuropsychologist | AWGS2 | The prevalence of MCI was significantly different according to sarcopenia status: the prevalence of MCI in sarcopenic adults was 80.6% vs in non-sarcopenic adults (61.6%) (p=0041). Sarcopenia was significantly associated with MCI (OR: 2.49 95% CI 1.23-5.05). However, when stratified by gender, sarcopenia was significantly associated with MCI in women (OR: 4.72 95% CI 1.39-15.97), whilst sarcopenia was not significantly associated with MCI in men (OR: 1.50 95% CI 0.57-3.96). |
| Cohort studies | | | | | | |
| Author, year | **Country** | **Population** | **Sample size** | **Assessment of cognition** | **Assesment of sarcopenia** | **Main results** |
| Beeri, 2021^16^ | USA | Community-dwelling older adults  Mean age: 81  % female subjects: 77.2% | 1175 | Diagnosed by neuropsychologist | EWGSOP2 | More severe sarcopenia was significantly associated with a higher risk of incident MCI after 5.6 years of follow-up (HR: 1.21 95%CI:1.01-1.45). |
| Hu, 2022^41^ | China | Community-dwelling: older adults (≥60years)  Mean age: 67.3  % female subjects: 43.8% | 5726 | Working Party of the International Psychogeriatric Association in collaboration with the World Health Organization | AWGS2 | The incidence of MCI after 3 years of follow-up was significantly different between the non-sarcopenic , possible sarcopenic and sarcopenic group (p<0.001): 10.1% for non-sarcopenia, 16.5% for possible sarcopenia, and 24.2% for sarcopenia. Possible sarcopenia and sarcopenia were significantly associated with the occurrence of MCI (possible sarcopenia: OR:1.43 95% CI: 1.06–1.91 and sarcopenia: OR: 1.72 95% CI: 1.04–2.85) when compared with the non-sarcopenic group. |
| Salinas-Rodriguez, 2021^66^ | Mexico | Community-dwelling older adults (≥50years)  Mean age: 65.5  % female subjects: 62.4 | 496 | NIA-AA | EWGSOP1 | The prevalence of MCI increased at an annual rate of 0.8% for non-sarcopenic older adults and nearly 1.5% for sarcopenic older adults after 8 years of follow-up. Sarcopenia was significantly associated with MCI (OR = 1.74 95% CI 1.02-2.96) |

| 1. Results from studies investigating the association between Alzheimer’s disease and sarcopenia | | | | | | |
| --- | --- | --- | --- | --- | --- | --- |
| Cross-sectional | | | | | | |
| Author, year | **Country** | **Population** | **Sample size** | **Assessment of cognition** | **Assesment of sarcopenia** | **Main results** |
| Bernard, 2016^20^ | USA | Community-dwelling AD patients (patient-caregiver dyads)  Mean age: 78  % female subjects: 54.6% | 168 | Diagnosed by physician in memory care practice (neuropsychological testing and clinical assessments) | SPSM | The mean SPSM score was 3,9 in patients with AD. There were no difference in SPSM scores across the MMSE quartiles. |
| Bramato, 2022^22^ | Italy | Community-dwelling AD patients  Mean age: 70.7  % female subjects: 54.51% | 130 | NIA-AA | EWGSOP1 & EWGSOP2 | 1) EWGSOP1-definition: The prevalence of sarcopenia was 23.8% in AD patients. 2) EWGSOP2-definition: The prevalence of sarcopenia was 4.6% in AD patients. |
| Cavazzotto, 2022^24^ | Brazil | Community-dwelling AD patients  Mean age: 77 (23 men-20 women  % female subjects: 45.45% | 43 | NINCDS-ADRDA | Low ASMI (Baumgartner) | 43.5% of the male AD patients had sarcopenia and 20% of the female AD patients had sarcopenia. |
| Chong, 2015^31^ | Singapore | Community-dwelling older adults (≥50years) and older adults attending the memory clinic with MCI or mild and moderate AD  Mean age: 70.8  % female subjects: 67.2% | 299 | NINCDS-ADRDA | AWGS1 | The prevalence of sarcopenia was significantly different dependent on cognitive status (p=0.000): 24.6% in healthy controls, 37.5% in MCI, 47.1% in mild AD and 60.0% in moderate AD. |
| Cintra, 2016^33^ | Brazil | Older patients with probable or possible Alzheimer's dementia in the community-geriatric clinic or emergency department (≥60years)    Mean age: 84.8  % female subjects: 85.1% | 67 | NINCDS-ADRDA | Calf circumference<31 | The prevalence of sarcopenia was 86.6% in AD patients. |
| Dost, 2022^17^ | Turkey | Hospitalized older adults in a Memory Clinic  Mean age: 73.6  % female subjects: 67.4% | 662 | NIA-AA | EWGSOP2 | The prevalence of sarcopenia was different dependent on cognitive status (p<0.001): The prevalence of probable sarcopenia and sarcopenia was 53.4% and 19.5%, respectively, in patients with AD. The prevalence of probable sarcopenia and sarcopenia was 55.9% and 19.1%, respectively, in patients with DLB. Probable sarcopenia, sarcopenia were significantly associated with AD (Probable sarcopenia; OR: 2.987 95%CI 1.805-4.944 and sarcopenia; OR: 3.723 95% CI 1.740-7.968). |
| Dost, 2022^35^ | Turkey | Older adults with probable AD admitted to geriatric clinic  Mean age: 76.6  % female subjects: 64.8% | 128 | NIA-AA | EWGSOP2 | The prevalence of probable and definitive sarcopenia was 54.7% and 18.7%, respectively in patients with AD. The prevalence of probable sarcopenia was significantly higher in those with CDR 2 than those with CDR 0.5 and 1 (p<0.002). |
| Gilette-Guyonnet, 2000^37^ | France | Hospitalized, but not institutionalized women with AD and non-institutionalized healthy women  Mean age: 81.6  % female subjects: 100% | 32 | NINCDS-ADRDA | Low ASMI (Baumgartner) | The prevalence of sarcopenia was not significantly different dependent on AD status: the prevalence of sarcopenia was 21.9% in healthy women without AD vs 40.6% in women with AD (p=0.10). Sarcopenia was not significantly associated with AD (OR: 2.44 95%CI:0.82-7.30). |
| Iritani, 2021^43^ | Japan | Community-dwelling older adults  Mean age: 79.5  % female subjects: 65.1% | 135 | DSM-5 | AWGS1 | The prevalence of sarcopenia was significantly different dependent on cognitive status (p<0.01): 25% in healthy subjects, 65.2% in subjects with MCI and 45.8% in subjects with AD. |
| Karim, 2022^45^ | Pakistan | AD male patients and healthy male controls  (68-82 years)  Mean age: 75  % female subjects: 0% | 203 | MMSE | EWGSOP2 | The prevalence of sarcopenia was significantly different dependent on cognitive status (p<0.05): The prevalence of sarcopenia was 20% in control subjects, 28% in subjects with early AD, 30% in subjects with mild AD and 38% in subjects with moderate AD. |
| Kimura, 2018^47^ | Japan | Community-dwelling patients with MCI and early-stage AD (65-89 years)  Mean age: 77.2  % female subjects: 63.4% | 205 | NIA-AA | AWGS1 | The prevalence of sarcopenia was 14.6% in patients with MCI and early-stage AD. |
| Lecheta, 2017^49^ | Brazil | Community-dwelling AD patients  Mean age: 78  % female subjects: 70.8% | 96 | Ministry of Health criteria | EWGSOP1 | The prevalence of pre-sarcopenia, sarcopenia and severe sarcopenia was 4.2%, 19.8% and 43.7%, respectively in older adults with AD. |
| Lee, 2020^51^ | Taiwan | Community-dwelling adults with mild to moderate AD  (65-89 years)  Mean age: 79.5  % female subjects: 68% | 125 | DSM-5 | EWGSOP1 | The prevalence of dementia was not significantly different dependent on sarcopenia status (p=0.288): The prevalence of mild dementia in sarcopenic older adults was 62.2% and the prevalence of moderate dementia in sarcopenic older adults was 37.8% vs. the prevalence of mild dementia was 72.7% in non-sarcopenic older adults and moderate dementia in non-sarcopenic older adults was 27.3%. |
| Liu, 2022^54^ | China | AD patients and healthy controls (60-85 years)  Mean age: 71.8  % female subjects: 68.3% | 82 | NIA-AA | AWGS2 | The prevalence of sarcopenia was significantly different dependent on cognitive status (p=0.035): normal cognition: 10.0%; mild AD: 25%; moderate AD: 38.9%. |
| Ogawa, 2018^61^ | Japan | Community-dwelling older adults with cardiometabolic diseases without severe CI  Mean age: 82  % female subjects: 77.2% | 285 | DSM-5 | AWGS1 | The prevalence of sarcopenia was significantly different dependent on cognitive status in male subjects (p<0.05): normal cognition: 13%; early AD: 41%; mild AD:47% and moderate AD: 47% and in female subjects (p<0.05): normal cognition:11%; early AD:36%; mild AD 45%; moderate AD: 60%. |
| Ozsureksi, 2020^62^ | Turkey | Community-dwelling probable AD patients  Mean age: 78.9  % female subjects: 56.4% | 76 | NINCDS-ADRDA | EWGSOP2 | The prevalence of sarcopenia were similar between the CDR stages in AD patients (p=0.20). In the probable sarcopenia group: CDR1=30.8%; CDR2= 48.5%; CDR3=63%. In the sarcopenia group: CDR1=15.4%; CDR22=6.5%; CDR 3=0%. In the severe sarcopenia group: CDR1= 15.4%; CDR2=13%; CDR 3: 21%. |
| Soysal, 2021^69^ | Turkey | Community-dwelling older adults with early stage AD or Lewy body dementia  Mean age: 81  % female subjects: 69.5% | 82 | NIA-AA | SARC-F | There were no significant differences in sarcopenia prevalence between DLB and AD (p=0.246). The prevalence of sarcopenia was 61.3% in DLB patients and 47.1% in AD patients. |
| Sugimoto, 2022^71^ | Japan | Community-dwelling adults and clinically diagnosed with MCI or AD (65-89 years)  Mean age: 77.7  % female subjects: 63.8% | 1181 | NIA-AA | Modified EWGSOP2: Asian-gender specific cut-off values for sarcopenia -defining parameters | The prevalence of sarcopenia was higher in subjects with AD than in subjects with MCI. AD: 30.2% (men) and 23.8% (women); MCI: 17.5% (men) and 12.7% (women). |
| Sugimoto, 2016^72^ | Japan | Community-dwelling adults attending the Memory Clinic (≥60years)  Mean age: 77.3  % female subjects: 66.8% | 418 | NINCDS-ADRDA | EWGSOP1 | The clinical stage of cognitive impairment was significantly different in sarcopenic older adults (p=0.047). The prevalence of normal cognition, MCI and AD in subjects with sarcopenia was 8.6%, 12.5% and 23.3%, respectively, whereas the prevalence of normal cognition, MCI and AD in subjects without sarcopenia was 91.4%, 87.5% and 76.7%, respectively. |
| Sugimoto, 2017^73^ | Japan | Community-dwelling AD patients  Mean age: 78  % female subjects: 64.9% | 208 | NIA-AA | AWGS1 | The prevalence of sarcopenia was 18.3% in AD patients. |
| Suzan, 2022^74^ | Turkey | Community-dwelling older adults (≥65years)  Mean age: 76.9  % female subjects: 71.7% | 339 | DSM-5 | EWGSOP2 | The prevalence of sarcopenia was significantly different dependent on AD status: the prevalence of sarcopenia was 52% in subjects with AD vs 28% in subject without AD (p<0.001). AD was significantly associated with sarcopenia (OR: 2.048 95% CI: 1.049–3.998). |
| Tay, 2018^77^ | Singapore | Community-dwelling older adults with MCI and mild to moderate AD (≥55years)  Mean age: 76.7  % female subjects: 64.8% | 108 | NINCDS-ADRDA | Modified EWGSOP1: Asian-gender specific cut-off values for sarcopenia -defining parameters | The prevalence of sarcopenia was not significantly different dependent on cognitive status (p=0.471): In subjects with MCI: 11.3% had sarcopenia and 28.6% had presarcopenia. In subjects with mild AD, 64.3% had sarcopenia and 69.8% had presarcopenia. In subjects with moderate AD, 18.9% had sarcopenia and 7.1% had presarcopenia. |
| Tsugawa, 2017^78^ | Japan | Community-dwelling older adults with probable AD  Mean age: 82.7  % female subjects: 59.3% | 218 | DSM-5 | AWGS1 | There were no significant differences in the prevalence of sarcopenia dependent on AD status. In women: AD/DM-: 38% ; AD/DM+:51%; DrD:50% and in men: AD/DM-:37%; AD/DM+:56% ; DrD: 50%. |
| Yazar, 2019^87^ | Turkey | Hospitalized patients with AD in neurology clinics and healthy controls aged 18-39 years old and 70-80 years old  Mean age:   - Healthy volunteers: 32.9 - AD patients: 75.7   % female subjects:   - Healthy volunteers: 21.5% - AD patients: 48.8% | 406 | NINCDS-ADRDA | EWGSOP1 | The prevalence of sarcopenia was significantly different between control group and AD patients (p<0.001): Control group: 13.8% had sarcopenia and AD group: 33.9% had sarcopenia. |
| Yildirim, 2021^89^ | Turkey | Community-dwelling AD patients (≥50years)  Mean age: 71.4  % female subjects: 58.1% | 630 | NIA-AA | Medical record: exact sarcopenia diagnosis not known | The prevalence of sarcopenia was 7.6% in patients with AD. Sarcopenia was only significantly associated with risk of having severe AD (OR: 7.41 95%CI 2.38-23.26). Sarcopenia was not significantly associated with risk of having moderate AD (OR: 2.52 95% CI: 0.86-7.41) or very severe AD (OR: 1.17 95%CI 0.12-11.36). |
| Zhang, 2022^93^ | China | Hospitalized patients (≥60years)  Mean age: 71.4  % female subjects: 39.3% | 168 | MoCA | AWGS2 | Among the patients, 20.8% had both sarcopenia and AD. |
| Takagi, 2017^75^ | Japan | AD patients requiring long-term care  Mean age: 85.4  % female subjects: 86.6% | 232 | DSM-3 and NINCD-ADRDA | Low ASMI (Baumgartner) | The prevalence of sarcopenia was 18.5% in AD patients. |
| Demura, 2023^34^ | Japan | Community-dwelling older adults with normal cognition to AD  Mean age: 80.9  % female subjects: 57.9% | 95 | DSM | AWGS2 | The prevalence of sarcopenia was significantly different dependent on cognitive status (p<0.05): 37.9% (normal cognition); 73.9% (MCI) and 62.8% (AD). |
| Liu, 2023^55^ | China | Community-dwelling AD female patients  Mean age: 72  % female subjects: 100% | 112 | NIA-AA and MMSE | AWGS2 | The prevalence of sarcopenia was 32.1% in AD patients. |
| Guner Oytun, 2023^38^ | Turkey | Community-dwelling adults with probable AD  Mean age: 76.82  % female subjects: 60.7% | 56 | NINCDS-ADRDA | EWGSOP2 | The prevalence of probable sarcopenia was 71.4% in AD patients. |
| Unsal, 2023^82^ | Turkey | Community-dwelling patients with AD  Mean age: 79.8  %female subjects: 58.1% | 253 | DSM-5 | EWGSOP2 | The prevalence of probable sarcopenia was 28.5% in AD patients. The prevalence of confirmed sarcopenia was significantly lower in mild AD group than in moderate AD group (16% vs 43.2%; p= 0.007). |
| Cohort studies | | | | | | |
| Author, year | **Country** | **Population** | **Sample size** | **Assessment of cognition** | **Assesment of sarcopenia** | **Main results** |
| Beeri, 2021^16^ | USA | Community-dwelling older adults  Mean age: 81  % female subjects: 77.2% | 1175 | NINCDS-ADRDA | EWGSOP2 | There was a significant difference in developing AD dependent on sarcopenia status: 70.0% of those with sarcopenia did develop AD, whereas 47.5% of those with sarcopenia did not develop AD (p<0.001). More severe sarcopenia was associated with a higher risk of incident AD after 5.6 years of follow-up (HR: 1.50 95%CI 1.20-1.86). |
| Interventional | | | | | | |
| Author, year | **Country** | **Population** | **Sample size** | **Assessment of cognition** | **Assesment of sarcopenia** | **Main results** |
| Yun, 2021^92^ | Korea | Hospitalized women with moderate-degree AD and sarcopenia admitted in nursing hospital (67-84 years)  Randomised study (12 week intervention: 5 training sessions on consecutive days (monday-friday) under supervision of a physiotherapist). Exercise consisted of kicking a ballon whilst lying in bed.  Mean age: 78.2  % female subjects: 100% | 26 | MMSE | Low ASMI  (Baumgartner) | There were no significant differences in SMI after 12 weeks of sessions in exercise group and control group (exercise group: difference in SMI = 0.01, p = 0.479; control group: difference in SMI = 0.01, p = 0.590) |

| 1. Results from studies investigating the association between other forms of dementia and sarcopenia | | | | | | |
| --- | --- | --- | --- | --- | --- | --- |
| Cross-sectional studies | | | | | | |
| Author, year | **Country** | **Population** | **Sample size** | **Assessment of cognition** | **Assesment of sarcopenia** | **Main results** |
| Calleja, 2019*^23^ | Spain | Hospitalized patients (≥75years) | 596 | Patient or caregiver interview | SARC-F | The prevalence of dementia was significantly different dependent on sarcopenia status: the prevalence was 36.2% in sarcopenic subjects vs 8.8% in non-sarcopenic subjects (p<0.001). |
| Chen, 2020^27^ | Taiwan | Hospitalized patients with hip fracture | 139 | SPMSQ | AWGS1 | The prevalence of dementia was significantly different dependent on sarcopenia status: 16.2% in sarcopenic older adults vs 10.0% in non-sarcopenic older adults (p=0.036). Dementia was not significantly associated with sarcopenia in patients with hip fracture (OR: 1.01 95% CI 0.22- 4.56). |
| Chen, 2022^29^ | Taiwan | Community-dwelling older adults with memory issues complaints (≥65years)  Mean age: 76.6  % female subjects:   - Sarcopenia group: 21% - Non-sarcopenia group: 30% | 81 | Consensus panel of neuropsychologists, neurologists and psychiatrists | AWGS2 | The prevalence of sarcopenia was not significantly different depending on dementia status (p=1.000): The prevalence of sarcopenia in patients with dementia and CDR 0.5 was 81.6% (sarcopenia group) and 83.7% (non-sarcopenia group); CDR 1: 13.2% (sarcopenia group) and 11.6% (non-sarcopenia group); CDR 2: 5.3% (sarcopenia group) ; 4.6% (non-sarcopenia group) |
| Cho, 2022^30^ | Korea | Community-dwelling older adults visiting the Memory Clinic and patients with dementia (≥60years)  Mean age: 77.9  % female subjects: 83.3% | 68 | NINCDS-ADRDA | Low ASMI (Baumgartner) | The prevalence of sarcopenia was 60.3% in patients with dementia. |
| Chou, 2022^32^ | Taiwan | Community-dwelling older adults with dementia (≥60years)  Mean age:76.5  % female subjects: 72.5% | 80 | Diagnosis of a neurologist or psychiatrist | AWGS2 | The prevalence of sarcopenia was 41.7% in patients with dementia. |
| Endo, 2021^36^ | Japan | Community-dwelling older adults (≥60years)  Mean age: 75  % female subjects: 65.6% | 753 | The Cognitive Assessment for Dementia | AWGS1 | The prevalence of dementia was significantly different dependent on sarcopenia status: 5.0% in non-sarcopenic older adults vs 9.6% in pre-sarcopenic older adults vs 23.3% in sarcopenic older adults (p<0.01). Dementia was not significantly associated with sarcopenia (OR: 1.65 95%CI 0.86-3.15). |
| Sperlich, 2021^70^ | Germany | Geriatric psychiatry hospitalized patients  Mean age: 78.9  % female subjects: 64.7% | 34 | ICD-10 classification  (F00-F03) | EWGSOP2 | The prevalence of sarcopenia was 65% in patients with dementia. |
| Tsugihashi, 2021^79^ | Japan | Community-dwelling older adults receiving home medical care  Mean age: 82.1  % female subjects: 61% | 181 | Medical record | SARC-F | Dementia was not significantly associated with sarcopenia (mean difference in SARC-F points of -0.35 95% CI:-1.15-0.45) |
| Umegaki, 2020^81^ | Japan | Community-dwelling older adults with early dementia and MCI  Mean age: 80.1  % female subjects: 54.1% | 74 | MMSE | AWGS2 | The prevalence of sarcopenia was 47% in patients with early dementia and MCI. |
| Yalcin, 2016^85^ | Turkey | Nursing home residents (≥65years)  Mean age: 79.17  % female subjects: 44% | 141 | Medical record | EWGSOP1 | The prevalence of sarcopenia in demented participants (58.5%) was significantly higher than the prevalence of sarcopenia in participants without dementia (38%) (p=0.041). |
| Yamada, 2021^86^ | Japan | Community-dwelling older adults at the Memory Clinic (≥65years)  Mean age: 77.5  % female subjects: 62.5% | 1774 | NIA-AA | Modified EWGSOP2: Asian-gender specific cut-off values for sarcopenia -defining parameters | The prevalence of both dementia (84.1%) and MCI (10.5%) were significantly higher in the sarcopenia group than in the non-sarcopenia group (dementia: 64.5% and MCI: 21%) (p<0.001). |
| Bianchi, 2017^21^ | Italy | Hospitalized older adults admitted to acute hospital ward  Mean age: 81  % female subjects: 51.9% | 655 | Medical record | EWGSOP1 | The prevalence of dementia was significantly different dependent on sarcopenia status: 1) in men: 8.5% in non-sarcopenic older adults vs 17.4% in sarcopenic older adults (p=0.018); 2) in women: 11.8% in non-sarcopenic older adults vs 25.9% in sarcopenic older adults (p=0.001). Sarcopenia was not significantly associated with dementia (OR:1.26 95% CI:0.93-1.70). |
| Cerri, 2015*^25^ | Italy | Hospitalized older adults admitted to acute hospital ward (≥65years)  Mean age: 84.2  % female subjects: 59.2% | 103 | Not specified | EWGSOP1 | The prevalence of dementia was not significantly different dependent on sarcopenia status: 31.8% in sarcopenic adults vs 20.7% in non-sarcopenic adults (p=0.11). |
| Sanchez-Castellano, 2020^67^ | Spain | Hospitalized patients with hip fracture (≥80years)  Mean age: 87.6  % female subjects: 78.7% | 150 | Global Deterioration Scale | Three used definitions: 1) EWGSOP2, 2) Janssen definition (low muscle mass), 3) Masanés definition (low muscle mass) | Patients with probable sarcopenia had more probability of having dementia : 1) Janssen definition: the prevalence of no dementia was 50.0%, mild dementia was 37.5%, moderate-severe dementia was 12.5% in sarcopenic older adults. 2) Masanés definition: the prevalence of no dementia was 60%, mild dementia was 25.7% and moderate-severe dementia was 14.3% in sarcopenic older adults. 3) EWGSOP2 definition: the prevalence of no dementia was 60.2%, mild dementia was 23.1% and moderate-severe dementia was 16.7% in sarcopenic older adults. |
| Peball, 2019^64^ | Italy | Community-dwelling older adults and patients with Parkinson's disease (≥65years)  Mean age: 73.8  % female subjects: 38.5% | 434 | Parkinon’s dementia: Patient interview | SARC-F | The prevalence of Parkinson’s dementia was 6.5% in sarcopenia group and 27.6% in non-sarcopenia group. Parkinsons' dementia was not significantly associated with sarcopenia (OR: 3.67 95%CI 0.85-15.81). |
| Landi, 2012^48^ | Italy | Nursing home residents (≥70years)  Mean age: 84.1  % female subjects: 75% | 122 | Medical record | EWGSOP1 | The prevalence of dementia was significantly different dependent on sarcopenia status: 47% in sarcopenic group and 67% in non- sarcopenic group. Dementia was not significantly associated with sarcopenia (OR: 0.31 95%CI 0.16-1.68). |
| Yigit, 2022^88^ | Turkey | Community-dwelling older adults (≥65years)  Mean age: 73.3  % female subjects: 55.7% | 201 | Standardized Mini-Mental test | EWGSOP2 | The prevalence of sarcopenia was significantly different dependent on cognitive status (p=0.006): 3.7% in normal cognition group vs in 15.4% in group with CI. |
| Dost, 2022^17^ | Turkey | Hospitalized older adults in a memory clinic  Mean age: 73.6  % female subjects: 67.4% | 662 | Fourth consensus report of the DLB Consortium | EWGSOP2 | The prevalence of probable sarcopenia and sarcopenia was 53.4% and 19.5%, respectively, in patients with AD. The prevalence of probable sarcopenia and sarcopenia was 55.9% and 19.1%, respectively, in patients with DLB. Probable sarcopenia were associated with DLB (OR: 2.52 95%CI 1.35–4.72, P=0.004) |
| Soysal, 2021^69^ | Turkey | Community-dwelling adults with early stage AD or Lewy body dementia  Mean age: 81  % female subjects: 69.5% | 82 | Fourth consensus report of the DLB Consortium | SARC-F | There were no significant differences in sarcopenia prevalence between DLB and AD (p=0.246). The prevalence of sarcopenia was 61.3% in DLB patients and 47.1% in AD patients. |
| Tamura, 2018^76^ | Japan | Community-dwelling adults with suggestive frailty symptoms from a special outpatient clinic of cardiology and diabetes  Mean age: 78  % female subjects: 62.2% | 323 | MMSE, Hasegawa’s Dementia Scale for Revised or Dementia Assessment Sheet in Community-based Integrated Care System-21 items | AWGS1 | 90% of subjects with sarcopenia had CI and 2.48% of the subjects had both sarcopenia and dementia. |
| Someya, 2022^68^ | Japan | Hospitalized older adults in a memory clinic  Mean age: 73.1  % female subjects: 57.6% | 1615 | MMSE | AWGS2 | Sarcopenia was not significantly associated with MCI (OR: 1.33 95% CI:0.92-1.93), but significantly associated with dementia (OR: 3.40 95% CI: 1.61-7.20). |
| Ülger, 2022^80^ | Turkey | Community-dwelling older adults (≥65years)  Mean age: 75.8  % female subjects: 64.7% | 221 | NIA-AA and DSM-5 | Two used definitions: 1) Low SMI and 2) Low sonographic gastrocnemius (GC) muscle thickness (via Ultrasound) combined with low HGS | The prevalence of sarcopenia-diagnosed by GC muscle thickness was higher in patients with dementia than those patients without dementia subjects (45.7% vs. 11.4%, p < 0.001). The prevalence of sarcopenia-diagnosed by SMI was higher in patients with dementia than those patients without dementia subjects (28.3% vs. 10.3%, p<0.001). Sarcopenia-diagnosed by GC muscle thickness was significantly associated with dementia (5.11 95%CI 2.00-13.03). |
| Chang, 2021^26^ | Taiwan | Older adults admitted to daycare centers (≥65years)  Mean age: 81.6  % female subjects: 75.0% | 173 | Medical record | AWGS2 | Dementia was significantly associated with confirmed sarcopenia (OR: 2.31 95%CI 1.05-5.04) |
| Cohort | | | | | | |
| Author, year | **Country** | **Population** | **Sample size** | **Assessment of cognition** | **Assesment of sarcopenia** | **Main results** |
| Yu, 2014^90^ | China | Community-dwelling older adults (≥65years)  Mean age: 72.5  % female subjects: 50% | 4000 | Community Screening instrument of dementia | EWGSOP1 | Probable dementia was not significantly associated with incident sarcopenia after 4 years of follow-up (OR: 0.98 95% CI 0.64-1.49). |
| Interventional | | | | | | |
| Author, year | **Country** | **Population** | **Sample size** | **Assessment of cognition** | **Assesment of sarcopenia** | **Main results** |
| Henwood, 2017^39^ | Australia | Institutionalized older adults with dementia  Interventional: non-randomised controlled trial study (12 week intervention for 2x/week). Exercise consisted of swimming for 45min.  Mean age: 82.4  % female subjects: 60.9% | 46 | Medical record | EWGSOP1 | Sarcopenia prevalence increased in both exercise and control group. However, participation to the exercise reduced the transition into sarcopenia. |

Abbreviations: MCI, mild cognitive impairment; AD, Alzheimer’s disease; HGS, handgrip strength; AWGS, Asian Working Group for Sarcopenia; EWGSOP, European Working Group in Sarcopenia in Older People; FNIH, Foundation for the National Institutes of Health definition; IWGS, International Working Group on Sarcopenia; SIG, Special Interest Group; SPSM, Short Portable Sarcopenia Measure; MMSE, Mini Mental State Examination; MoCA, Montreal Cognitive Assessment, TYM, Test Your Memory; SPMSQ, Short Portable Mental Status Questionnaire; NIA-AA, National Institute on Aging—Alzheimer’s Association; NINCDS-ADRDA, National Institute of Neurological and Communicative Disorders and Stroke and the Alzheimer’s Disease and Related Disorders Association; DSM, Diagnostic and Statistical Manual of Mental Disorders; DLB, Lewy Body Dementia; ICD, International Classification of Diseases; CDR, Clinical Dementia Rating; (A)SMI, (appendicular) skeletal muscle mass index; GC, gastrocnemius; DM, Diabetes Mellitus; DrD, Diabetes-related Dementia; OR, odds ratio; CI, confidence interval. *: Cohort study, but a cross-sectional analysis was used to explore the prevalence of dementia in sarcopenic older adults.

**Supplementary Material 3a: Quality assessment of cross-sectional studies using the modified Newcastle-Ottawa scale**

| **Author, year** | **Selection**  **(***)** | **Comparability**  **(**)** | **Outcome**  **(**)** | **Total** |
| --- | --- | --- | --- | --- |
| Bai, 2021 | * | ** | * | **** |
| Bernard,2016 | * | - | * | ** |
| Bramato, 2022 | - | - | * | * |
| Calleja, 2019 | * | - | * | ** |
| Cavazzotto, 2022 | - | ** | * | *** |
| Chen, 2021 | * | ** | * | **** |
| Chang, 2021 | * | - | ** | *** |
| Chen, 2020 | * | - | * | ** |
| Chen, 2022 | * | - | * | ** |
| Cho,2022 | - | - | * | * |
| Chong, 2015 | ** | - | * | *** |
| Chou, 2022 | * | - | * | ** |
| Cintra, 2016 | * | - | * | ** |
| Dost, 2022 | * | ** | * | **** |
| Dost, 2022 | * | - | * | ** |
| Endo, 2021 | * | ** | * | **** |
| Gilette-Guyonnet, 2000 | * | - | * | ** |
| Ida, 2017 | - | ** | * | *** |
| Iritani, 2021 | * | ** | - | *** |
| Jacob, 2021 | ** | - | * | *** |
| Karim, 2022 | * | - | * | ** |
| Kim, 2014 | * | ** | * | **** |
| Kimura, 2018 | * | ** | * | **** |
| Lecheta, 2017 | * | - | * | ** |
| Lee, 2020 | * | ** | * | **** |
| Lee, 2018 | * | ** | * | **** |
| Liu, 2022 | * | - | * | ** |
| Liu, 2020 | ** | ** | * | ***** |
| Machii, 2020 | * | ** | * | **** |
| Oba, 2022 | * | ** | * | **** |
| Ogama, 2019 | * | ** | * | **** |
| Ogawa, 2018 | * | - | * | ** |
| Ozsureksi, 2020 | * | - | * | * |
| Papachristou, 2015 | ** | ** | - | **** |
| Someya, 2022 | * | ** | * | **** |
| Soysal, 2021 | * | - | * | ** |
| Sperlich, 2021 | * | - | - | * |
| Sugimoto, 2022 | * | - | * | ** |
| Sugimoto, 2016 | * | - | * | ** |
| Sugimoto, 2017 | * | - | * | ** |
| Suzan, 2022 | * | - | * | ** |
| Tamura, 2018 | * | - | * | ** |
| Tay, 2018 | * | - | * | ** |
| Tsugawa, 2017 | * | - | * | ** |
| Tsugihashi, 2021 | - | ** | ** | **** |
| Umegaki, 2020 | * | - | - | * |
| Woo, 2015 | * | - | * | ** |
| Yalcin, 2016 | * | - | * | ** |
| Yamada, 2021 | * | - | * | ** |
| Yazar, 2019 | * | - | - | * |
| Yildirim, 2021 | - | - | * | * |
| Yuenyongchaiwat, 2020 | * | ** | * | **** |
| Zhang, 2022 | * | - | * | ** |
| Bianchi, 2017 | * | ** | * | **** |
| Cerri, 2015 | * | - | * | ** |
| Hu, 2021 | * | ** | * | **** |
| Li,2022 | * | ** | * | **** |
| Rodrigues-Rejon, 2020 | ** | ** | * | ***** |
| Sanchez-Castellano, 2020 | * | - | - | * |
| Peball, 2019 | * | ** | * | **** |
| Landi, 2012 | * | ** | * | **** |
| Takagi, 2017 | * | - | * | ** |
| Demura, 2023 | * | - | * | ** |
| Liu, 2023 | * | - | * | ** |
| O'Donovan, 2022 | ** | ** | - | **** |
| Yigit, 2022 | * | - | * | ** |
| Guner Oytun, 2023 | * | - | * | ** |
| Wang, 2022 | * | - | * | ** |
| Unsal, 2023 | * | - | * | ** |
| Lee, 2023 | * | ** | * | **** |
| Ülger, 2022 | * | ** | * | **** |

**Supplementary Material 3b: Quality assessment of cohort studies using the Newcastle-Ottawa scale**

| **Author, year** | **Selection**  **(****)** | **Comparability**  **(**)** | **Outcome**  **(***)** | **Total**  **(9)** |
| --- | --- | --- | --- | --- |
| Beeri, 2021 | **** | ** | ** | ******** |
| Hu, 2022 | **** | ** | * | ******* |
| Salinas-Rodriguez, 2021 | ** | ** | * | ***** |
| Yu, 2014 | *** | ** | ** | ******* |

**Supplementary Material 3c: Quality assessment of interventional studies**

1. Non-randomized studies of interventions (ROBINS-I tool)

| **Author, year** | **Bias due to confounding** | **Bias in selection of participants into the study** | **Bias in classification of interventions** | **Bias due to deviations from intended interventions** | **Bias due to missing data** | **Bias in measurement of outcomes** | **Bias in selection of reported results** | **Overall risk of bias** |
| --- | --- | --- | --- | --- | --- | --- | --- | --- |
| Henwood, 2017 | Low | Serious | Low | Low | Moderate | Serious | Low | Serious |

1. Randomized studies of interventions (RoB 2)

| **Author, year** | **Domain 1: Randomisation proces** | **Domain 2:**  **Deviations from the intended interventions** | **Domain 3:**  **Missing outcome data** | **Domain 4:**  **Measurement of the outcome** | **Domain 5: Selection of the reported results** | **Overall risk of bias** |
| --- | --- | --- | --- | --- | --- | --- |
| Yun, 2021 | Low | High | Low | Low | Low | Low |

**Supplementary Material 4: Sensitivity analyses**

1. Correlation between mild cognitive impairment and sarcopenia

| **Study omitted** | **Estimate (95%CI)** |
| --- | --- |
| Bai (2021) | 0.450 (0.340-0.559) |
| Chen (2021) | 0.452 (0.342-0.563) |
| Hu (2022) | 0.451 (0.342-0.562) |
| Hu (2022) | 0.471 (0.355-0.586) |
| Ida (2017) | 0.448 (0.340-0.556) |
| Jacob (2021) | 0.446 (0.325-0.565) |
| Jacob (2021) | 0.463 (0.354-0.572) |
| Jacob (2021) | 0.442 (0.331-0.553) |
| Jacob (2021) | 0.460 (0.351-0.568) |
| Jacob (2021) | 0.458 (0.351-0.566) |
| Jacob (2021) | 0.457 (0.349-0.564) |
| Kim (2014) | 0.449 (0.341-0.556) |
| Lee (2018) | 0.447 (0.340-0.555) |
| Lee (2018) | 0.450 (0.342-0.558) |
| Liu (2020) | 0.480 (0.362-0.599) |
| Machii (2020) | 0.458 (0.349-0.565) |
| Papachristou (2015) | 0.457 (0.349-0.565) |
| Papachristou (2015) | 0.454 (0.346-0.562) |
| Salinas-Rodriguez (2021) | 0.451 (0.341-0.561) |
| Someya (2022) | 0.471 (0.359-0.583) |
| Yuenyongchaiwat (2020) | 0.465 (0.358-0.573) |
| Lee (2023) | 0.445 (0.336-0.553) |
| Combined | 0.456 (0.348-0.563) |

1. Correlation between Alzheimer’s disease and sarcopenia

Meta-analyses estimates, given named study is omitted

| **Study omitted** | **Estimate (95%CI)** |
| --- | --- |
| Dost (2022) | 1.036 (0.683-1.390) |
| Dost (2022) | 1.081 (0.667-1.496) |
| Suzan (2022) | 1.197 (0.833-1.561) |
| Yildirim (2021) | 1.102 (0.768-1.436) |
| Yildirim (2021) | 1.009 (0.676-1.342) |
| Yildirim (2021) | 1.106 (0.783-1.428) |
| Combined | 1.087 (0.767-1.406) |

1. Correlation between non-AD dementia and sarcopenia

Meta-analyses estimates, given named study is omitted

| **Study omitted** | **Estimate (95%CI)** |
| --- | --- |
| Chen (2020) | 0.379 (0.182-0.577) |
| Endo (2021) | 0.361 (0.156-0.566) |
| Bianchi (2017) | 0.476 (0.220-0.733) |
| Peball (2019) | 0.356 (0.159-0.554) |
| Landi (2012) | 0.417 (0.219-0.615) |
| Yu (2014) | 0.483 (0.262-0.704) |
| Dost (2022) | 0.314 (0.108-0.520) |
| Someya (2022) | 0.311 (0.109-0.514) |
| Ülger (2022) | 0.316 (0.116-0.516) |
| Combined | 0.373 (0.178-0.569) |

**Supplementary Material 5: Funnel plot for publication bias**

1. Correlation between mild cognitive impairment and sarcopenia


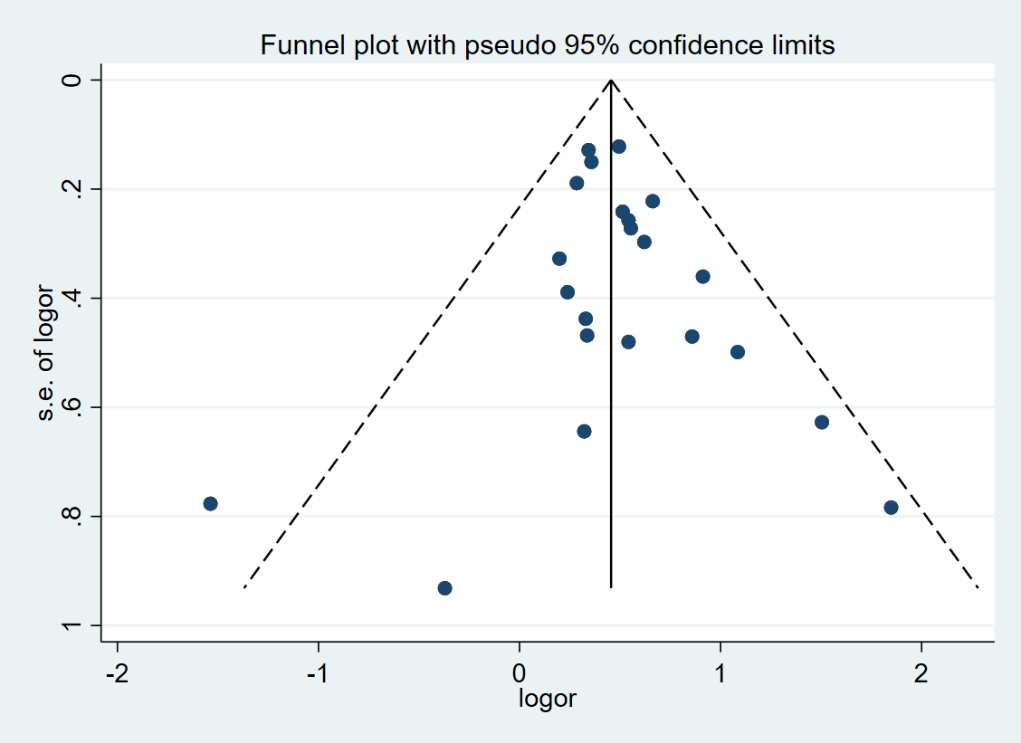


1. Correlation between Alzheimer’s disease and sarcopenia


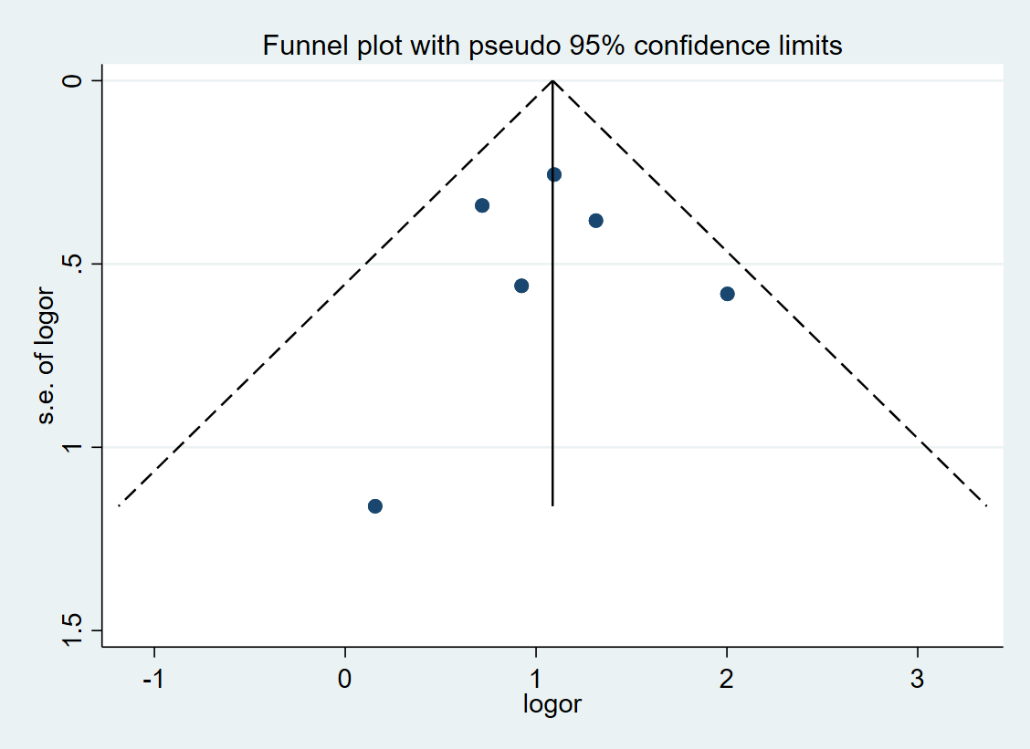


1. Correlation between non-AD dementia and sarcopenia


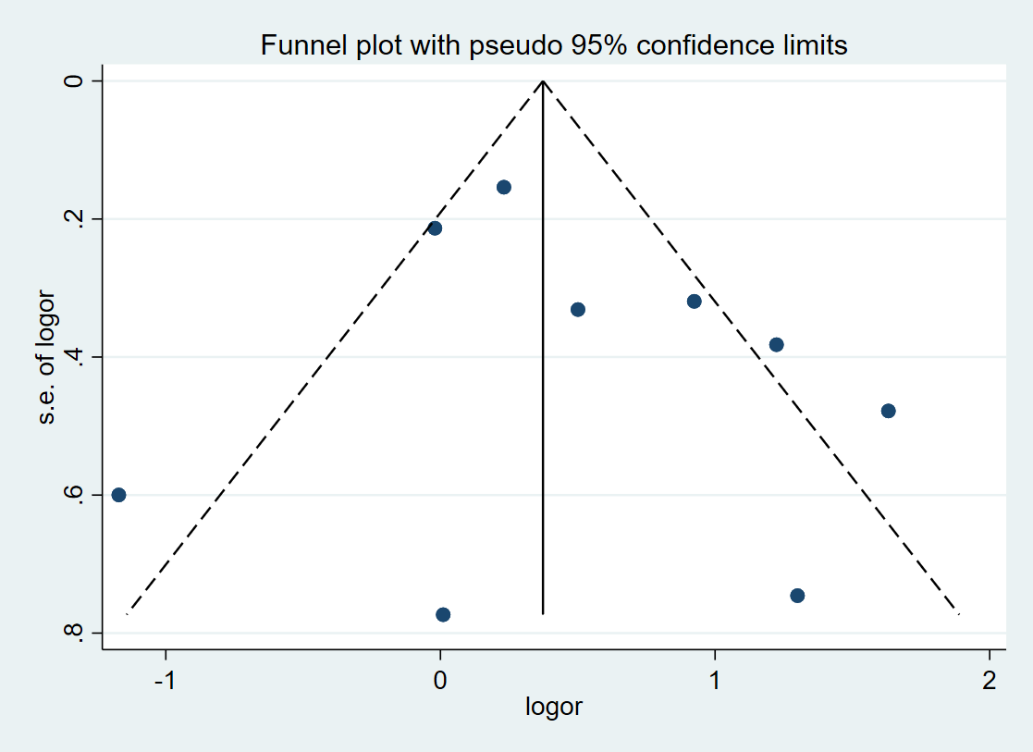


**Supplementary Material 6: Egger’s test for publication bias**

| **Egger’s test** | Intercept | SE | P |
| --- | --- | --- | --- |
| Correlation MCI and sarcopenia | 0.305 (-0.586-1.197) | 0.427 | 0.483 |
| Correlation AD and sarcopenia | 0.028 (-3.126-3.183) | 1.136 | 0.981 |
| Correlation other types of dementia and sarcopenia | 1.024 (-0.067-2.115) | 0.535 | 0.065 |

| **Section and Topic** | **Item #** | **Checklist item** | **Location where item is reported** |
| --- | --- | --- | --- |
| **TITLE** | | |  |
| Title | 1 | Identify the report as a systematic review. | Title page |
| **ABSTRACT** | | |  |
| Abstract | 2 | See the PRISMA 2020 for Abstracts checklist. | Page 2-3 |
| **INTRODUCTION** | | |  |
| Rationale | 3 | Describe the rationale for the review in the context of existing knowledge. | Page 5 |
| Objectives | 4 | Provide an explicit statement of the objective(s) or question(s) the review addresses. | Page 6 |
| **METHODS** | | |  |
| Eligibility criteria | 5 | Specify the inclusion and exclusion criteria for the review and how studies were grouped for the syntheses. | Page 7 |
| Information sources | 6 | Specify all databases, registers, websites, organisations, reference lists and other sources searched or consulted to identify studies. Specify the date when each source was last searched or consulted. | Page 7 |
| Search strategy | 7 | Present the full search strategies for all databases, registers and websites, including any filters and limits used. | Supplementary material 1 |
| Selection process | 8 | Specify the methods used to decide whether a study met the inclusion criteria of the review, including how many reviewers screened each record and each report retrieved, whether they worked independently, and if applicable, details of automation tools used in the process. | Page 7 |
| Data collection process | 9 | Specify the methods used to collect data from reports, including how many reviewers collected data from each report, whether they worked independently, any processes for obtaining or confirming data from study investigators, and if applicable, details of automation tools used in the process. | Page 7 |
| Data items | 10a | List and define all outcomes for which data were sought. Specify whether all results that were compatible with each outcome domain in each study were sought (e.g. for all measures, time points, analyses), and if not, the methods used to decide which results to collect. | Page 7 |
|  | 10b | List and define all other variables for which data were sought (e.g. participant and intervention characteristics, funding sources). Describe any assumptions made about any missing or unclear information. | Page 7 |
| Study risk of bias assessment | 11 | Specify the methods used to assess risk of bias in the included studies, including details of the tool(s) used, how many reviewers assessed each study and whether they worked independently, and if applicable, details of automation tools used in the process. | Page 8 |
| Effect measures | 12 | Specify for each outcome the effect measure(s) (e.g. risk ratio, mean difference) used in the synthesis or presentation of results. | Page 8 |
| Synthesis methods | 13a | Describe the processes used to decide which studies were eligible for each synthesis (e.g. tabulating the study intervention characteristics and comparing against the planned groups for each synthesis (item #5)). | Page 8 |
|  | 13b | Describe any methods required to prepare the data for presentation or synthesis, such as handling of missing summary statistics, or data conversions. | Page 8 |
|  | 13c | Describe any methods used to tabulate or visually display results of individual studies and syntheses. | Page 8 |
|  | 13d | Describe any methods used to synthesize results and provide a rationale for the choice(s). If meta-analysis was performed, describe the model(s), method(s) to identify the presence and extent of statistical heterogeneity, and software package(s) used. | Page 8 |
|  | 13e | Describe any methods used to explore possible causes of heterogeneity among study results (e.g. subgroup analysis, meta-regression). | Page 8 |
|  | 13f | Describe any sensitivity analyses conducted to assess robustness of the synthesized results. | Page 8 |
| Reporting bias assessment | 14 | Describe any methods used to assess risk of bias due to missing results in a synthesis (arising from reporting biases). | Page 8 |
| Certainty assessment | 15 | Describe any methods used to assess certainty (or confidence) in the body of evidence for an outcome. | Page 8 |
| **RESULTS** | | |  |
| Study selection | 16a | Describe the results of the search and selection process, from the number of records identified in the search to the number of studies included in the review, ideally using a flow diagram. | Figure 1 |
|  | 16b | Cite studies that might appear to meet the inclusion criteria, but which were excluded, and explain why they were excluded. | Figure 1 |
| Study characteristics | 17 | Cite each included study and present its characteristics. | Page 9-11 and Supplementary material 2 |
| Risk of bias in studies | 18 | Present assessments of risk of bias for each included study. | Supplementary material 3 |
| Results of individual studies | 19 | For all outcomes, present, for each study: (a) summary statistics for each group (where appropriate) and (b) an effect estimate and its precision (e.g. confidence/credible interval), ideally using structured tables or plots. | Page 12-13 , Figure 2-4, Table 1-3 and Supplementary material 2 |
| Results of syntheses | 20a | For each synthesis, briefly summarise the characteristics and risk of bias among contributing studies. | Page 11 |
|  | 20b | Present results of all statistical syntheses conducted. If meta-analysis was done, present for each the summary estimate and its precision (e.g. confidence/credible interval) and measures of statistical heterogeneity. If comparing groups, describe the direction of the effect. | Page 12-13 , Figure 2-4, Table 1-3 |
|  | 20c | Present results of all investigations of possible causes of heterogeneity among study results. | Page 12-13 , Figure 2-4, Table 1-3 |
|  | 20d | Present results of all sensitivity analyses conducted to assess the robustness of the synthesized results. | Supplementary material 4 |
| Reporting biases | 21 | Present assessments of risk of bias due to missing results (arising from reporting biases) for each synthesis assessed. | Supplementary material 4 |
| Certainty of evidence | 22 | Present assessments of certainty (or confidence) in the body of evidence for each outcome assessed. | Page 12-13 , Figure 2-4, Table 1-3 |
| **DISCUSSION** | | |  |
| Discussion | 23a | Provide a general interpretation of the results in the context of other evidence. | Page 14-16 |
|  | 23b | Discuss any limitations of the evidence included in the review. | Page 14-16 |
|  | 23c | Discuss any limitations of the review processes used. | Page 14-16 |
|  | 23d | Discuss implications of the results for practice, policy, and future research. | Page 16-18 |
| **OTHER INFORMATION** | | |  |
| Registration and protocol | 24a | Provide registration information for the review, including register name and registration number, or state that the review was not registered. | Page 7 |
|  | 24b | Indicate where the review protocol can be accessed, or state that a protocol was not prepared. | Page 7 |
|  | 24c | Describe and explain any amendments to information provided at registration or in the protocol. | NA |
| Support | 25 | Describe sources of financial or non-financial support for the review, and the role of the funders or sponsors in the review. | Page 19 |
| Competing interests | 26 | Declare any competing interests of review authors. | Page 19 |
| Availability of data, code and other materials | 27 | Report which of the following are publicly available and where they can be found: template data collection forms; data extracted from included studies; data used for all analyses; analytic code; any other materials used in the review. | NA |

*From:*  Page MJ, McKenzie JE, Bossuyt PM, Boutron I, Hoffmann TC, Mulrow CD, et al. The PRISMA 2020 statement: an updated guideline for reporting systematic reviews. BMJ 2021;372:n71. doi: 10.1136/bmj.n71

For more information, visit: <http://www.prisma-statement.org/>
